# Dynein light chain regulates adaptive and innate B cell development by distinctive genetic mechanisms

**DOI:** 10.1371/journal.pgen.1007010

**Published:** 2017-09-18

**Authors:** Ashleigh King, Lingli Li, David M. Wong, Rui Liu, Rebecca Bamford, Andreas Strasser, David M. Tarlinton, Jörg Heierhorst

**Affiliations:** 1 Molecular Genetics Unit, St. Vincent's Institute of Medical Research, Fitzroy, Victoria, Australia; 2 Department of Medicine (St. Vincent’s Health), University of Melbourne, Fitzroy, Victoria, Australia; 3 Molecular Genetics of Cancer Division, The Walter and Eliza Hall Institute, Parkville, Victoria, Australia; 4 Department of Medical Biology, University of Melbourne, Parkville, Victoria, Australia; 5 Department of Immunology and Pathology, Monash University, Melbourne, Victoria, Australia; The Jackson Laboratory, UNITED STATES

## Abstract

Mechanistic differences in the development and function of adaptive, high-affinity antibody-producing B-2 cells and innate-like, “natural” antibody-producing B-1a cells remain poorly understood. Here we show that the multi-functional dynein light chain (DYNLL1/LC8) plays important roles in the establishment of B-1a cells in the peritoneal cavity and in the ongoing development of B-2 lymphoid cells in the bone marrow of mice. Epistasis analyses indicate that *Dynll1* regulates B-1a and early B-2 cell development in a single, linear pathway with its direct transcriptional activator ASCIZ (ATMIN/ZNF822), and that the two genes also have complementary functions during late B-2 cell development. The B-2 cell defects caused by loss of DYNLL1 were associated with lower levels of the anti-apoptotic protein BCL-2, and could be supressed by deletion of pro-apoptotic BIM which is negatively regulated by both DYNLL1 and BCL-2. Defects in B cell development caused by loss of DYNLL1 could also be partially suppressed by a pre-arranged *SW*_*HEL*_ *Igm*-B cell receptor transgene. In contrast to the rescue of B-2 cell numbers, the B-1a cell deficiency in *Dynll1*-deleted mice could not be suppressed by the loss of *Bim*, and was further compounded by the *SW*_*HEL*_ transgene. Conversely, oncogenic MYC expression, which is synthetic lethal with *Dynll1* deletion in B-2 cells, did not further reduce B-1a cell numbers in *Dynll1*-defcient mice. Finally, we found that the ASCIZ-DYNLL1 axis was also required for the early-juvenile development of aggressive MYC-driven and p53-deficient B cell lymphomas. These results identify ASCIZ and DYNLL1 as the core of a transcriptional circuit that differentially regulates the development of the B-1a and B-2 B lymphoid cell lineages and plays a critical role in lymphomagenesis.

## Author summary

Antibody-producing B cells can be segregated into two major populations: The better known conventional B-2 cells typically produce high-affinity and mono-specific antibodies, but only after they encounter a particular pathogen or in response to vaccines. In contrast, the B-1a cells constitutively produce lower-affinity broad-specificity “natural” antibodies that serve as a preemptive defense against a wide range of microbes. Here we reveal that the transcription factor ASCIZ and its target DYNLL1 are essential for mice to have a normally sized pool of B-1a cells in place shortly after birth. We show that these two factors function in a single linear pathway during the development of B-1a cells. This interaction represents a rare example where the activity of a transcription factor, in this case ASCIZ, can be explained by the effects of a single target gene, in this case *Dynll1*. While ASCIZ and DYNLL1 are also required for producing normal numbers of B-2 cells, we discovered that they regulate B-1a cells and B-2 cells by distinct genetic mechanisms. Finally, we found that ASCIZ also contributes to the early onset of B-1a B cell-derived lymphoid cancers in juvenile mice. The results provide insight into the development of an important cell population of the immune system.

## Introduction

B lymphoid cells are essential for the humoral immune response by producing a diverse range of antigen-specific antibodies. Antibody-mediated immunity is provided by two distinctive B cell lineages that diverge early in life. The better-known conventional, or B-2, B cells provide adaptive immunity by producing high-affinity pathogen-specific antibodies, typically in a T cell-dependent manner. The pool of B-2 cells is constantly replenished from hematopoietic stem and progenitor cells in the bone marrow [1]. B-1a cells provide innate-like immunity by producing “natural” low-affinity, broad-specificity antibodies against a wide range of overlapping antigens, including self-antigens. In contrast to B-2 cells, the pool of B1-a cells is largely established at birth and these cells are able to self-renew as mature cells in the periphery [2, 3].

During their differentiation in the bone marrow, B-2 lineage precursors go through a series of well-defined developmental stages [1, 4]. Critical events are the rearrangement of the immunoglobulin (Ig) heavy chain-encoding *Igh* locus through V(D)J recombination at the pro-B cell stage (Hardy fraction B); association of this unique IgH with a generic surrogate Ig light chain (λ5 and v-preB) to form the pre-B cell receptor (pre-BCR) in pre-B cells (fraction C); pre-BCR-dependent clonal expansion by ~5 cell division cycles during the large pre-B cell stage (fraction C’) [5]; VJ recombination of the *Igλ/κ* light chain (IgL) loci during the small pre-B cell stage (fraction D); and association of IgH and IgL chains to form an IgM complex/B cell receptor (BCR) at the immature B cell stage (fraction E). The immature B cell stage represents a critical quality control phase during which cells with high-affinity self-reactive BCRs are eliminated through BIM-dependent apoptosis [6]. Immature B cells that pass this quality control stage exit the bone marrow, and undergo further maturation in peripheral lymphoid tissues, such as the spleen and lymph nodes. There, upon stimulation by cognate antigens and T cell help, activated B cells can again enter the cell cycle, alternating with further diversification of the V(D)J-rearranged *Ig* loci through somatic hyper-mutation of *Ig* variable regions and class-switch recombination of *Igh* constant regions.

Whereas adult B lymphopoiesis in the bone marrow almost exclusively produces B-2 cells, B-1a cells are derived from fetal stem cells, and B-1a precursors dominate early B cell development in the fetal liver and early post-natal spleen [2, 3, 7]. Thus, in mice, B-1a lineage cells constitute the major B cell compartment until ~3 weeks of age [8, 9]. In adults, B-1a cells are less numerous than B-2 cells and most reside in the peritoneal cavity, where they also can undergo further AID-mediated antibody diversification by somatic hyper-mutation and Ig class-switch recombination, albeit in a stochastic, age-dependent manner that appears to be independent of exogenous antigens [10]. B-2 and B-1a cells differ in their transcriptional programs as well as growth factor dependence [3, 11], and they have markedly different Ig repertoires [2]. In particular, B-1a pools seem to be biased towards expressing V(D)J-rearranged IgH chains that associate only poorly with the surrogate light chain [2]. Although the understanding of the functional differences between B-1a and B-2 cells is continuously increasing, the developmental mechanisms that underlie these differences still remain poorly understood.

The Zn^2+^-finger protein ASCIZ (also known as ATMIN [12, 13]), which functions as a highly specific transcription factor for the multifunctional dynein light chain, DYNLL1 (also known as LC8) [14–16], plays critical roles in B-2 cell development [13, 17] and B cell lymphomagenesis [18]. DYNLL1 is a common subunit of the cytoplasmic, intra-flagellar and axonemal Dynein motor complexes [19–22], but also binds numerous Dynein-independent targets [23, 24], including the apoptosis initiating BH3-only protein BIM [25]. We have previously shown that ectopic expression of DYNLL1 could rescue the severe defect in B cell development caused by the absence of ASCIZ, confirming that defective regulation of DYNLL1 plays a key role in the defects in B lymphopoiesis caused by *Asciz* deficiency.

Based on its established role in the B cell defects observed in ASCIZ-deficient mice, we sought to directly investigate the role of DYNLL1 during B cell development. We show here that conditional deletion of *Dynll1* largely phenocopies the B-2 cell developmental defects seen in *Asciz*-deleted mice, although the two genes function in part by different mechanisms. We also show that the ASCIZ-DYNLL1 axis is particularly critical for the development of B-1a cells. Surprisingly, the B-1a and B-2 cell deficiencies in *Dynll1*-deleted mice were differentially affected by genetic modifiers, indicating that DYNLL1 regulates the development of the two B cell lineages via distinct genetic mechanisms.

## Results

### DYNLL1 is required for B-2 cell homeostasis in mice

**Fig 1:**
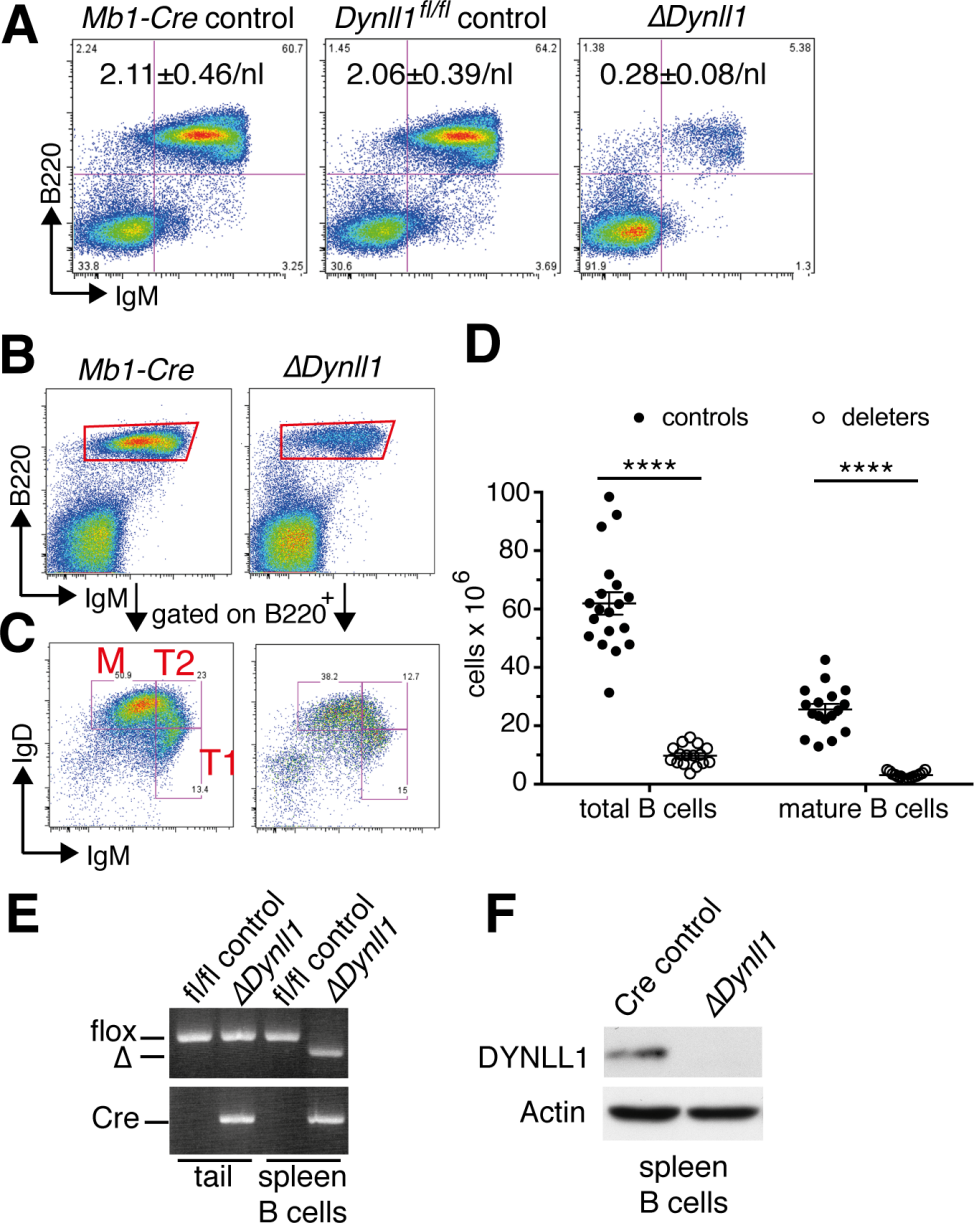
Reduced B cell numbers in *Mb1-Cre Dynll1*-deleted mice. (A) FACS analysis of peripheral blood cells from 4-week-old mice with the genotypes indicated above. Numbers indicate mean ± S.E. of 2 independent experiments (n = 4 for *Mb1-Cre* control, n = 3 for *Dynll1*^*fl/fl*^ control, n = 4 for *ΔDynll1*). (B-C) Representative FACS plots and gating strategies to determine total and mature splenic B cell numbers. (D) Summary of splenic B cell numbers from >3 independent experiments (n≥15 per group). (E) PCR genotyping of tail DNA and MACS-purified splenic B cell DNA. (F) Western blot analysis of extracts from purified splenic B cells.

To investigate the role of DYNLL1 in B cell development, we deleted a conditional floxed *Dynll1* allele (*Dynll1*^*fl*^) using the *Mb1-Cre* knock-in allele, which is efficiently expressed from the late pre-pro-B cell stage onwards [26]. Peripheral blood cell analyses at 4 weeks-of-age revealed a severe depletion of circulating B cells in *Mb1-Cre*^*ki/+*^ *Dynll1*^*fl/fl*^-deleted mice compared to *Mb1-Cre*^*ki/+*^ or *Dynll1*^*fl/fl*^ controls (Fig 1A). Similar lymphopenia was observed in the spleens of 8-week-old *Mb1-Cre*^*ki/+*^ *Dynll1*^*fl/fl*^-deleted mice, with an >8-fold reduction of mature B cells numbers (Fig 1B–1D). Although transitional B cells, which give rise to both follicular and marginal zone B cells, were reduced in the spleens of *Dynll1*-deleted mice (Fig 1C), only the follicular B cell numbers were affected by the absence of DYNLL1 (S1 Fig), presumably because marginal zone B cells have a much longer half-life than follicular B cells [27]. Genotyping and Western blot analyses of MACS-purified splenic B cells from *Mb1-Cre*^*ki/+*^ *Dynll1*^*fl/fl*^ mice confirmed the efficient deletion of the targeted *Dynll1* alleles, and complete loss of DYNLL1 protein (Fig 1E and 1F). These results indicate that DYNLL1 is required for normal B cell production in mice.

### DYNLL1 is required for the proliferation and survival of developing B-2 linage cells in the bone marrow

**Fig 2:**
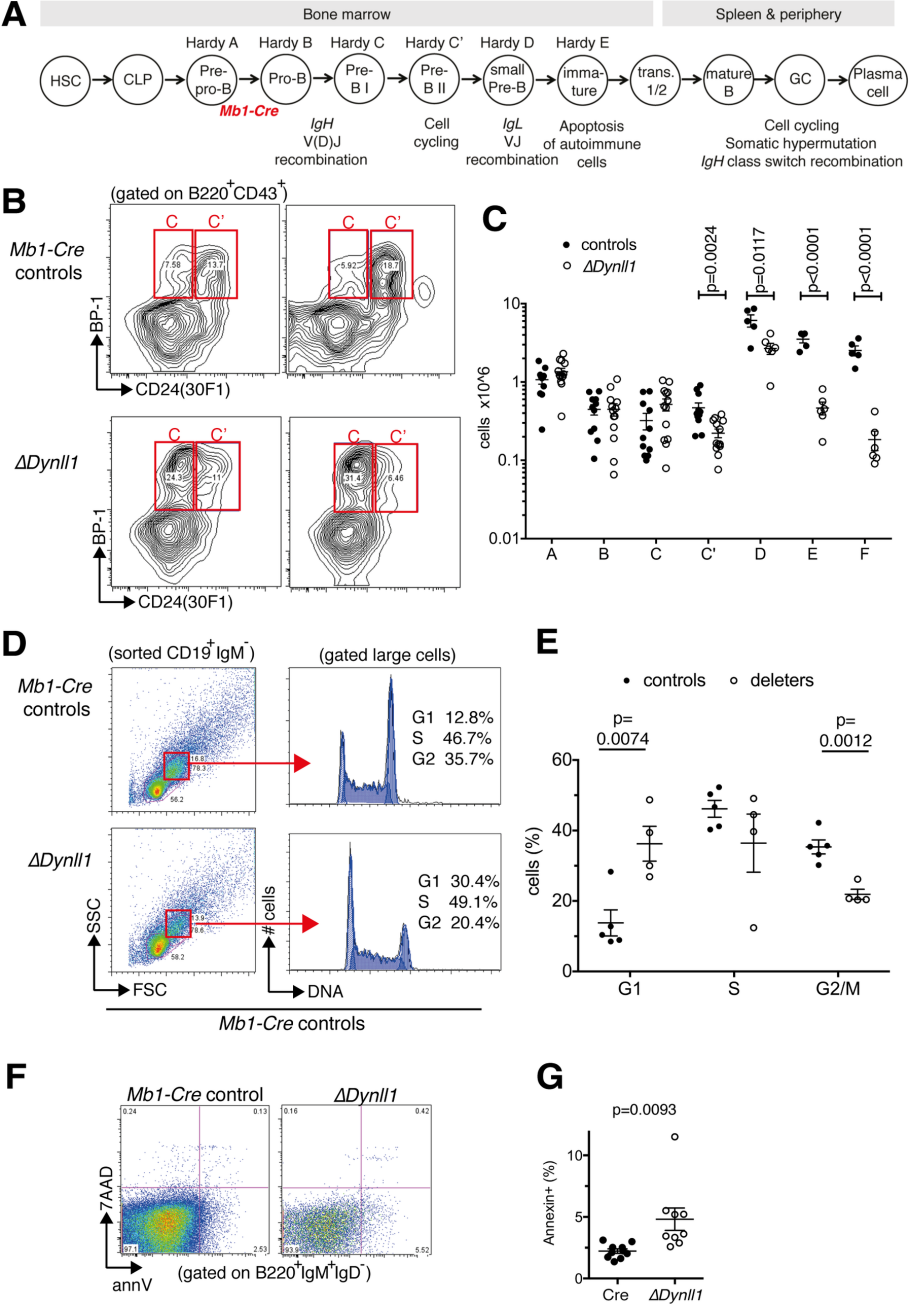
Defect in bone marrow B cell development in *Mb1-Cre Dynll1*-deleted mice. All mice were analyzed at 8 weeks of age. (A) Schematic diagram of B cell developmental stages. (B) FACS plots of B220^+^CD43^+^ bone marrow cells from 2 *Mb1-Cre* control mice and *2 Dynll1*-deleted mice with fractions C and C’ highlighted. Related FACS plots for fractions D-E are shown in S2 Fig. (C) Summary data of >3 independent experiments (n = 5–11 per group). (D, E) Cell cycle distribution of large pre-B cells. Representative FACS plots and gating strategies (D), and grouped data (mean ± S.E.) of 3 independent experiments (n = 4–5 per group). (F, G) Representative FACS plots and grouped data (mean ± S.E.) of apoptotic cells in the immature B cell fraction E (n = 9–10; >3 independent experiments).

To determine how loss of DYNLL1 leads to reduced peripheral B-2 cell numbers, we monitored B cell development in the bone marrow of 8-week-old mice using surface marker staining according to Hardy [5] (Fig 2). There were no differences during the first two stages (fractions B and C) following *Mb1-Cre* mediated recombination of the floxed *Dynll1* alleles, but loss of DYNLL1 led to a >2-fold reduction of the cycling pre-B cell pool (fraction C’) (Fig 2B and 2C). This difference was maintained during the small (post-cycling) pre-B cell stage (fraction D), and was further exacerbated to an 8-to-10-fold reduction of immature B cells (fraction E) and recirculating B cells (fraction F) in *Dynll1*-deleted mice compared to *Mb1-Cre* controls (Fig 2C and S2 Fig). These data reveal that DYNLL1 has critical functions during two distinct developmental stages: the cycling pre-B cell stage and the immature B cell stage.

To explore how loss of DYNLL1 lead to reduced cell numbers at these two stages, we performed cell cycle analyses of fraction C' cells (which represents the only developmental stage during which B-2 cell precursors proliferate [5]) and viability analyses of fraction E cells. FACS-sorted pro-B and pre-B cells (CD19^+^ IgM^-^) were stained for DNA content analysis, and gated on the larger cycling pre-B cells (Fig 2D). This analysis revealed a >2.5-fold increased proportion of pre-B cells in G_0_/G_1_ phase in *Dynll1*-deleted mice compared to the control animals, and a corresponding reduction of S and G2/M phase cells (Fig 2D and 2E). Moreover, even though *Dynll1*-deleted mice contained ~10-fold fewer immature B cells, the proportion of apoptotic cells within this fraction was increased by >2-fold compared to the *Mb1-Cre* control animals (Fig 2F and 2G). There were no significant differences in the cell surface levels of the pre-BCR or BCR in early pre-B cells or immature B cells, respectively (S3 Fig). This indicates that the developmental defects at these stages are not due to altered pre-BCR or BCR expression. These results indicate that loss of DYNLL1 impairs pre-B cell proliferation to a large extent by delaying them in G1 phase, and that the additional cell loss at the immature B cell stage is due to increased apoptotic cell death.

### Defective B cell development in *Dynll1*-deleted mice can be suppressed by loss of pro-apoptotic BIM but not by loss of p53

**Fig 3:**
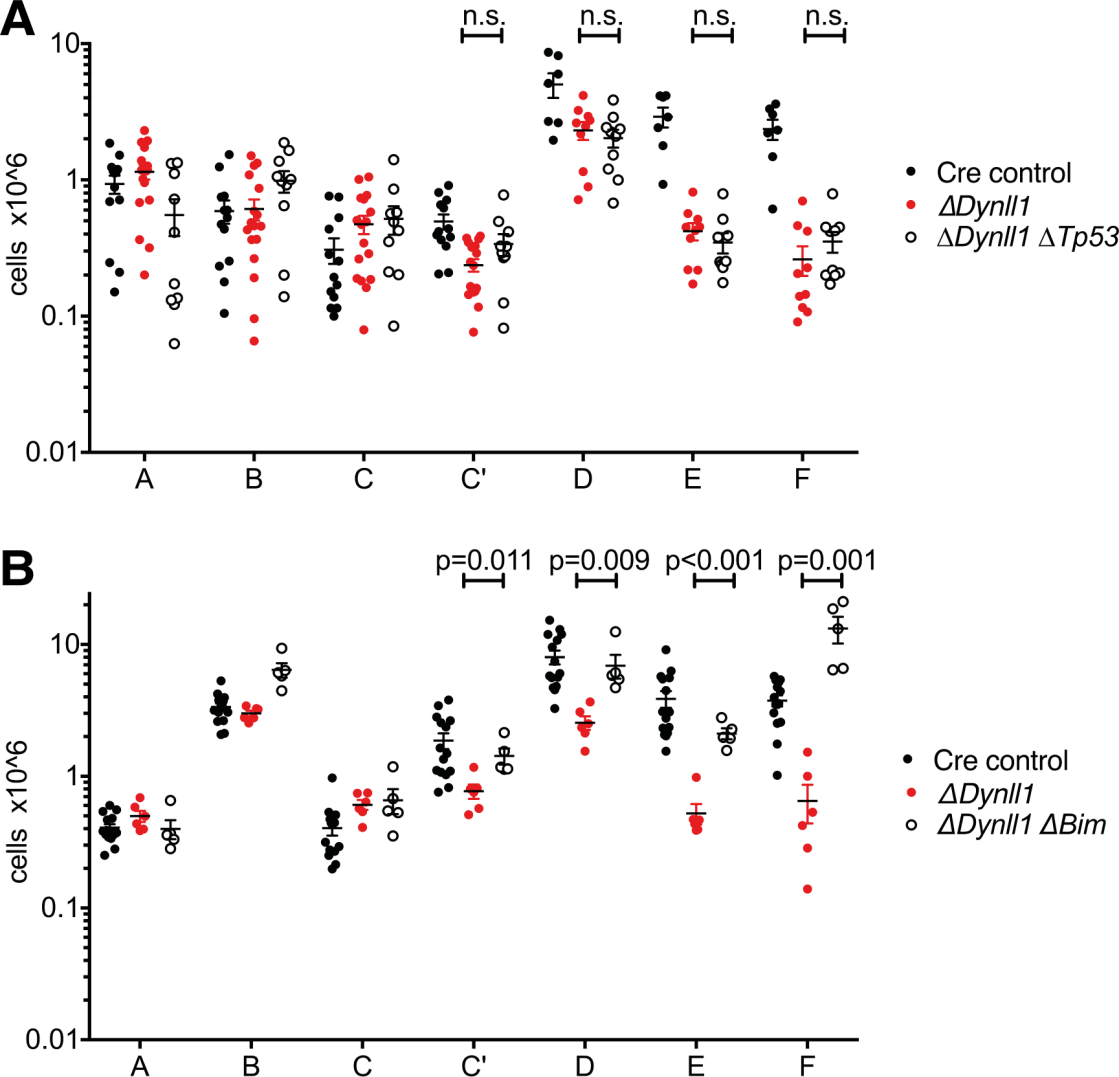
B cell development defects in *Dynll1*-deleted mice are rescued by deletion of *Bim* but not by loss of p53. (A) Effect of concomitant *tp53* deletion. Data points for *Mb1-Cre* controls and *Dynll1*-deleted mice include the same mice shown in panel C, plus additional mice for both groups that were analyzed in parallel with the *Mb1-Cre Dynll1 tp53* compound mutant mice (n = 10) in >3 independent experiments. (B) Effect of concomitant *Bim* deletion. Different *Mb1-Cre* control and *ΔDynll1* animals from those in Figs 2C and 3A were analyzed in parallel with *Dynll1-/Bim*-double deleted mice (n = 5–15) in >3 independent experiments.

To evaluate possible reasons for the G1 phase delay in pre-B cells and the increased apoptosis of immature B cells caused by loss of DYNLL1, we performed additional gene deletion studies. Increased proportions of cells in G1 phase are often the result of a p53-mediated checkpoint arrest, for example in response to endogenous or exogenously induced DNA damage, or mitotic chromosome separation defects during the previous cell cycle [28]. To test this hypothesis, we additionally deleted the *Tp53* gene in these mice. However, concomitant conditional deletion of *Tp53* did not increase the numbers of cycling fraction C’ cells (and subsequent fraction D and E cells) in *Mb1-Cre Dynll1*-deleted mice (Fig 3A). This demonstrates that the delayed cell cycling was not a consequence of a p53-mediated cell cycle arrest.

The pro-apoptotic BH3-only protein BIM plays key roles in the survival of B lymphoid cells during multiple stages of their development, for example in response to limiting concentrations of cytokines [29, 30] and in the negative selection against self-reactive BCRs [6]. To test if BIM, which is known to be negatively regulated by DYNLL1 through direct binding, was involved in the B cell development defects of *Dynll1*-deleted mice, we also deleted *Bim* in these cells. Remarkably, co-deletion of *Bim* completely restored the number of fraction C' cells, as well as the subsequent fraction D and E cells, in the absence of DYNLL1 back to the amounts found in *Mb1-Cre* control mice (Fig 3B)[note that the increased numbers of recirculating B cells (fraction F) cells are a consequence of abnormally increased peripheral B cell numbers, a characteristic of *Bim* knockout mice [6, 17, 31]].

Collectively, these results indicate that the numerical aberrations at different stages of B cell development of *Dynll1*-deficient mice, are predominantly driven by BIM-dependent apoptosis but independent of p53.

### Loss of DYNLL1 leads to reduced expression of BCL-2 in B lymphoid cells

**Fig 4:**
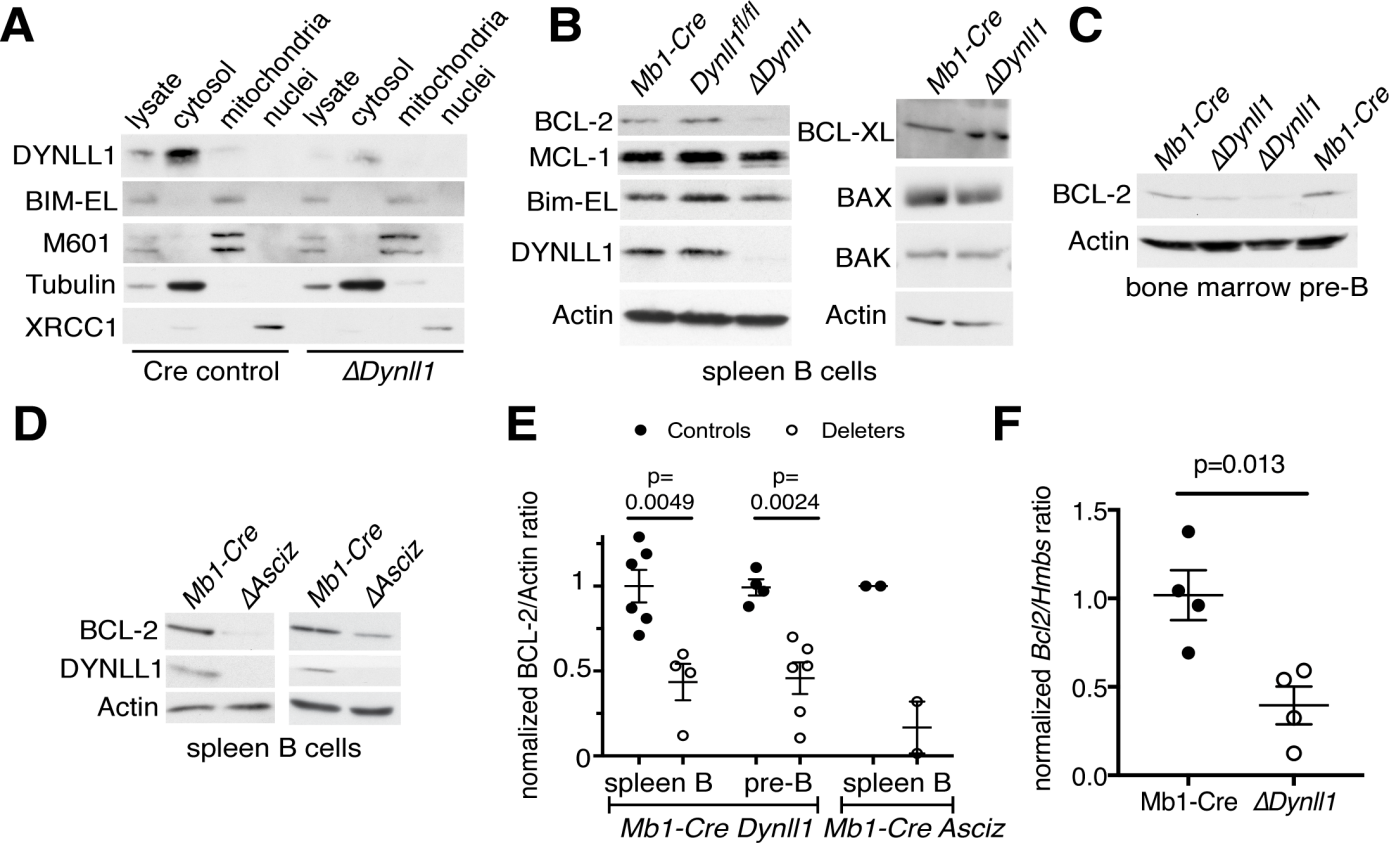
Loss of DYNLL1 leads to reduced BCL-2 expression in B lymphoid cells. (A) Subcellular fraction assays of DYNLL1 and BIM in purified mature B cells. M601 serves as a marker for mitochondria, tubulin for cytosol and microtubuli, and XRCC1 for nuclei. (B-D) Western blot analyses of the indicated proteins in extracts of MACS-purified splenic B cells (B, D) or FACS-purified bone marrow pre-B cells (C, B220^+^ CD43^-^ IgM^-^). (E) Quantification of relative BCL-2 protein levels in splenic B cells and bone marrow pre-B cells from control and *Asciz*-deleted or *Dynll1*-deleted mice as indicated. (F) Quantification of relative *Bcl2* mRNA levels in MACS-purified B cells from *Mb1-Cre* control and *Dynll1*-deleted mice.

To determine how deletion of *Bim* may rescue B cell development in the absence of DYNLL1, we measured the expression of BIM and other BCL-2 family proteins in *Dynll1*-deleted mice (Fig 4). The total levels of BIM protein, and its subcellular distribution, were not altered in *Dynll1*-deleted B cells compared to control cells (Fig 4A and 4B). Unexpectedly, we found that expression of the BIM antagonist BCL-2 was consistently and significantly reduced by ~2-fold in both mature and developing *Dynll1*-deleted B lineage cells compared to cells from control animals (Fig 4B, 4C and 4E). This finding was independently confirmed in B cells that lack the *Dynll1*-transcription factor ASCIZ, and consequently contain only very low amounts of DYNLL1 (Fig 4D). Finally, reverse transcription PCR analyses indicated that the expression of *Bcl2* was reduced at the mRNA level (Fig 4F).

These data indicate that DYNLL1 is required for efficient *Bcl2* expression and to restrain BIM-mediated apoptosis in developing B cells.

### ASCIZ and DYNLL1 act in a single linear pathway at the pre-B cell stage but have overlapping and additive functions during the immature B cell stage

**Fig 5:**
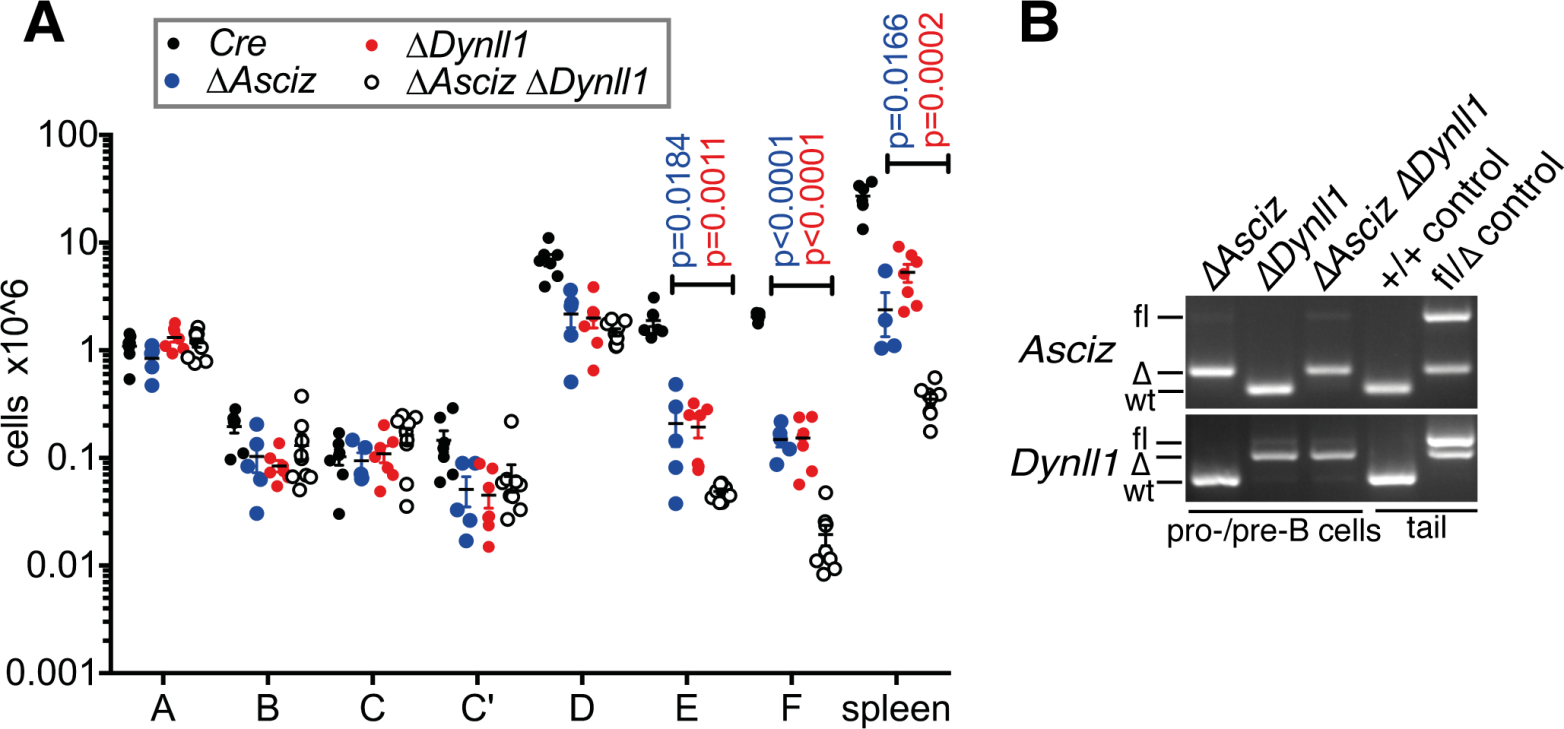
Comparison of B-2 cell developmental defects in *Dynll1*-deleted, *Asciz*-deleted and *Asciz/Dynll1*-double deleted mice. (A) Grouped data of >3 independent experiments with mice of all four genotypes (n = 5–9). The *Mb1-Cre* control, and *Dynll1*-deleted mice are different from those shown in Fig 2. P values indicate differences between *Dynll1*-deleted mice (red) and *Asciz*-deleted mice (blue) compared to double mutant animals. Representative FACS plots used for the quantifications are shown in S4 Fig. (B) PCR genotyping of *Asciz* and *Dynll1* deletion efficiency in the pooled FACS-sorted (B220^+^ CD19^+^ IgM^-^) pro-B and pre-B cell stages (fractions B-D).

The relative cell losses at the pre-B and immature B cell stages in *Dynll1*-deleted mice were reminiscent of what we have previously reported for mice with a B cell-specific conditional deletion of ASCIZ, the critical transcriptional regulator of *Dynll1* [17]. Thus, to determine the extent to which these two genes function in the same pathway, we examined additional cohorts of *Mb1-Cre Dynll1*-deleted and *Mb1-Cre Asciz*-deleted, as well as *Mb1-Cre Asciz/Dynll1*-double-deleted mice for side-by-side comparisons of B-2 cell development. Interestingly, this analysis showed that loss of either *Dynll1* or *Asciz* caused quantitatively almost identical effects on B cell development from fraction C’ onwards (Fig 5A). Moreover, compared to the single mutants, the reduction in the cycling fraction C’ cells (which is carried over to the subsequent small pre-B stage, fraction D) was not further increased in *Asciz/Dynll1* double mutants compared to the single mutant animals. This demonstrates that ASCIZ regulates the proliferation of cycling pre-B cells entirely through DYNLL1.

In contrast to their epistatic interaction at the cycling pre-B cell stage, we found that concomitant deletion of both genes led to a further 5-to-10-fold depletion of immature B cells (fraction E), recirculating mature B cells (fraction F) and splenic B cells compared to the single knockout animals (Fig 5A and S4 Fig). This increased loss of immature B cells in the double knockout mice indicates that ASCIZ and DYNLL1 must also have some non-overlapping functions at this later stage of B-2 cell development. Thus, depending on the specific stage of B cell development, ASCIZ and DYNLL1 can act in a single linear pathway (pre-B cells), or they can also have additional functions independently of each other (immature B cells).

### B-2 lymphopenia in *Dynll1*-deleted mice can be rescued partially by transgenic expression of a rearranged B cell receptor

The more severe phenotype of the *Asciz/Dynll1* double mutant animals (which completely lack DYNLL1) compared to the *Asciz* single mutants (which still retain ~5–10% of normal levels of DYNLL1 [14, 17, 32]) indicated that even very low residual amounts of DYNLL1 can sustain some of its functions either in immature B cells, or during the transition from the small pre-B to the immature B cell stage. As the level of apoptosis of immature B cell was comparable between *Dynll1*-deleted (Fig 2G) and *Asciz*-deleted mice [17], we hypothesized that the low remaining DYNLL1 levels in *Asciz* mutants may have a specific role in the generation, rather than the survival, of immature B cells. As the generation of immature B cells (which are defined by the expression of a complete BCR) depends on the successful recombination of the *Igλ/κ* loci during the small pre-B cell stage (fraction D), we tested whether complementation with a pre-arranged BCR transgene could restore the numbers of immature B cells in *Mb1-Cre Dynll1*-deleted mice.

**Fig 6:**
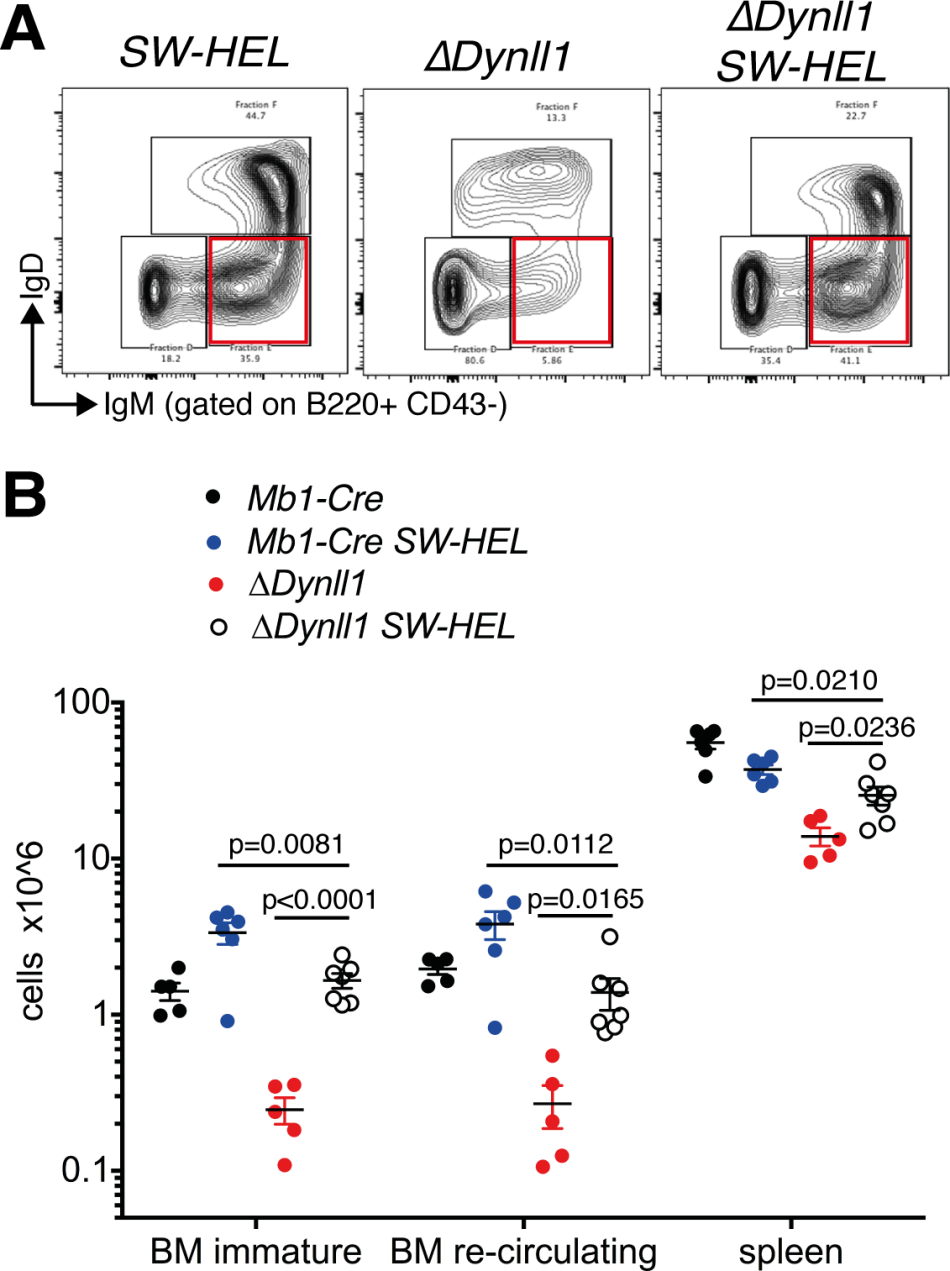
Partial rescue of the B-2 lymphopenia in *Dynll1*-deleted mice by the pre-arranged *SW*_*HEL*_ BCR. (A) Representative FACS plots of fraction D-F cells (B220^+^ CD43^-^) with immature B cells (IgM^+^ IgD^-^) highlighted. (B) Grouped data of ≥3 independent experiments (n = 5–7 per group). *SW*_*HEL*_ mice are homozygous for the *IgV*_*H*_*10* heavy chain knock-in allele, and contain a *V*_*κ*_*10* light chain transgene(s).

Remarkably, the anti-hen egg lysozyme-specific *SW*_*HEL*_ *Igh* knock-in and *Igl* transgene [33] restored the numbers of immature B cells in *Mb1-Cre Dynll1*-deleted mice to a level comparable to those seen in *Mb1-Cre* control mice (Fig 6A and 6B), although they did not quite reach the abnormally elevated levels of immature B cells in *Mb1-Cre SW*_*HEL*_ control mice (presumably because the chicken-specific *SW*_*HEL*_ BCR does not activate auto-reactive apoptotic programs in mice). It should be noted that the *SW*_*HEL*_ transgene did not increase the numbers of immature B cells in *Mb1-Cre Asciz*-deleted mice [17]. Thus, the specific rescue effect of the pre-arranged BCR for *Mb1-Cre Dynll1*-deleted mice supports the notion that DYNLL1 may play an ASCIZ-independent role in some aspect of *Igl* gene rearrangement or BCR surface expression.

### Loss of DYNLL1 or ASCIZ leads to severe peripheral B-1a lymphopenia

**Fig 7:**
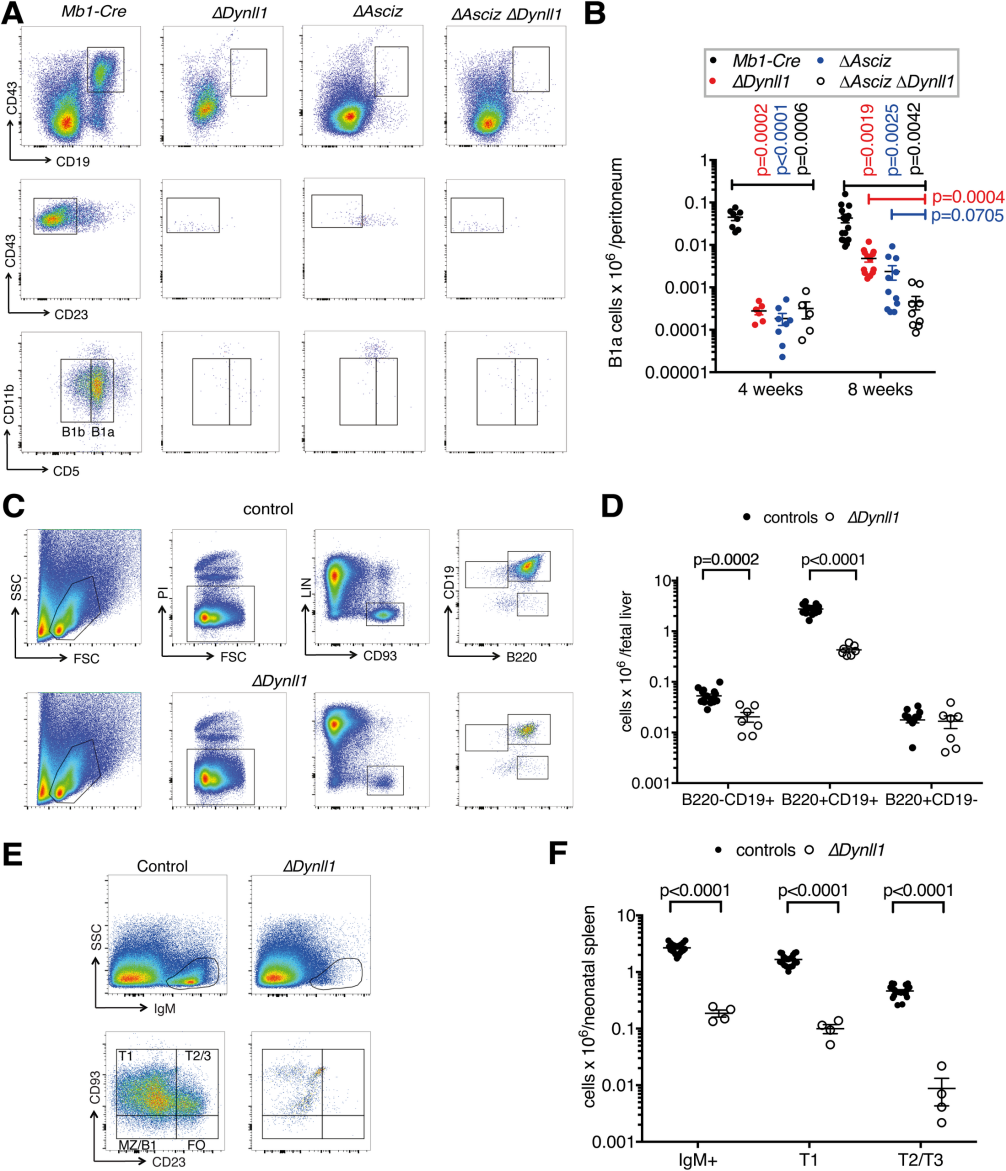
DYNLL1 is required for normal B-1a cell development. (A, B) Representative FACS plots and gating strategies and grouped data of peritoneal cavity cells of 4-week (A, B) and 8-week (B) old mice from ≥3 independent experiments (n = 5–16 per group). P values indicate differences of *Dynll1*-deleted (red), *Asciz*-deleted (blue) and *Dynll1/Asciz* double-deleted mice (black) compared to *Mb1-Cre* controls. Representative FACS plots for 8-week-old mice are shown in S5 Fig. (C, D) Representative FACS plots and gating strategies of B cell progenitors in E17.5 fetal livers. B-1a progenitors (B220^-^ CD19^+^), B-2 progenitors (B220^+^ CD19^-^) and B-1a/-2 precursors (B220^+^ CD19^+^) are boxed. Data are from 3 independent experiments using plugged *Mb1-Cre*^*Ki/+*^ *Dynll1*^*fl/+*^ X *Mb1*^*+/+*^ *Dynll1*^*fl/fl*^ matings (n = 7–16 per group). The control group includes *Mb1-Cre*^*Ki/+*^ *Dynll1*^*fl/+*^, *Mb1*^*+/+*^ *Dynll1*^*fl/+*,^ and *Mb1*^*+/+*^ *Dynll1*^*fl/fl*^ mice. (E, F) Representative FACS plots and gating strategies and grouped data for splenocytes from 3 independent experiments (n = 4–20 per group) using 4–5 day old mice using the same breeding strategy as in panels C-D.

Next, we quantified B-1a cells in 4-week-old *Dynll1*-deleted, *Asciz*-deleted and *Asciz/Dynll1*-double deleted mice. At this age, B-1a cell pools are established in the peritoneal cavity and thereafter expand through self-renewal [2, 3, 7]. Remarkably, in all three mutant strains, B-1a cell numbers in the peritoneal cavity were reduced by >100-fold compared to age-matched controls (Fig 7A and 7B). This demonstrates that the ASCIZ-DYNLL1 axis is crucial for the development of the initial B-1a cell pool.

Peritoneal B-1a cells are derived from precursors in the fetal liver that transition through the spleen during the early post-natal period. In fetal livers at day E17.5, B-1a progenitors (Lin^-^ CD93^+^ B220^-/low^ CD19^+^)[34] were reduced by ~2.5-fold in *Dynll1*-deleted embryos compared to control littermates (Fig 7C and 7D), while early B-2 progenitors were present at normal levels (Fig 7C and 7D). In the fetal livers of *Dynll1*-deleted embryos there was also an ~6-fold reduction in the more prominent Lin^-^ CD93^+^ B220^int^ CD19^+^ fraction, which most likely represents more differentiated B-1a precursors (Fig 7C and 7D). Interestingly, at 4–5 days of age, the spleens of *Dynll1*-deleted mice contained >20-fold fewer transitional B cells, which at this stage primarily represent B-1 lineage cells [8], than littermate controls (Fig 7E and 7F). Overall, these results indicate that the defects resulting in the impaired production of B-1a lineage cells in the absence of DYNLL1 start at the progenitor stage in the fetal liver and become more severe around the neonatal transitional stage, when these cells first become surface IgM-positive. As all three mutant mouse strains had a quantitatively identical defect in establishing the initial B-1a cell pools at 4 weeks of age, we assume that *Asciz*-deleted and *Asciz/Dynll1*-double deleted mice would have comparable B-1a progenitor and transitional cell deficiencies to *Dynll1*-deleted embryos and newborns.

It should be noted that between 4 and 8 weeks of age, peritoneal B-1a cell numbers increased considerably in *Dynll1* and *Asciz* single mutants, presumably through accelerated self-renewal, but interestingly, they did not increase in the *Asciz/Dynll1*-double mutants (Fig 7B and S5 Fig). These results reveal that ASCIZ and DYNLL1 function in a single linear pathway that is essential for the initial establishment of the B-1a cell pool, but they may have some partially independent functions during the subsequent self-replicating expansion of B-1a cells.

### Differential rescue of the B-2 but not B-1a lymphopenia of *Dynll1*-deleted mice by the *SW*_*HEL*_ B cell receptor transgene or loss of *Bim*

As shown above, the B-2 lymphopenia in *Dynll1*-deleted mice could be ameliorated by expression of the pre-arranged *SW*_*HEL*_ BCR knock-in/transgene or by deletion of pro-apoptotic *Bim*. We therefore also investigated how these genetic modifiers affect the B-1a cell lineage in these mutant mice.

**Fig 8:**
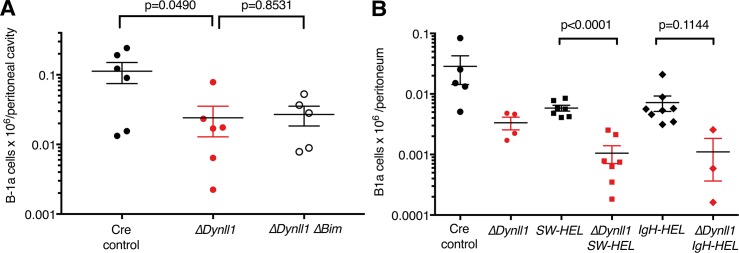
*Bim* loss and *SW*_*HEL*_ BCR transgene expression do not rescue the B-1a lymphopenia in *Dynll1*-deleted mice. (A) B1-a cells were quantified in the peritoneal cavities at 8 weeks of age in the same mice as shown in Fig 3B. (B) B-1a cells were quantified in 8-week old mice (including those shown in Fig 6) in ≥3 independent experiments. *SW*_*HEL*_ mice contain the complete HEL BCR (homozygous *IgV*_*H*_*10* heavy chain knock-in plus *V*_*κ*_*10* light chain transgene), *IgH-HEL* mice contain the homozygous *IgV*_*H*_*10* knock-in but lack the Ig light chain transgene.

Surprisingly, in contrast to its pronounced rescue effect on B-2 cell numbers (Fig 3B), deletion of *Bim* had no protective effect on peritoneal B-1a cells in DYNLL1-deficient mice (Fig 8A and S6 Fig). The numbers of B-1a cells in *Dynll1*-deleted mice were also not increased, but rather further reduced by expression of the *SW*_*HEL*_ transgene (Fig 8B).

As indicated above, B-1a and B-2 cells differ markedly in their Ig repertoires [2], and the V_H_10/V_*κ*_10 BCR encoded by *SW*_*HEL*_ represents a classical high-affinity antibody characteristic of affinity matured conventional B-2 lymphocytes [33]. Consistent with this notion, the *SW*_*HEL*_ BCR, or just its (homozygous) *V*_*H*_*10* heavy chain knock-in, led to a ~5-fold reduction of B-1a cells compared to control mice, and thereby further compounded the B-1a lymphopenia in *Dynll1*-deleted mice (Fig 8B and S6 Fig). Nevertheless, the specific rescue of B-2 cells but not B-1a cells by these two genetic modifiers indicates that *Dynll1* regulates B-1a and B-2 cell development via distinct, lineage-specific mechanisms.

### Differential synthetic lethal interaction between loss of *Dynll1* and oncogenic MYC expression in B-2 but not B-1a cells

**Fig 9:**
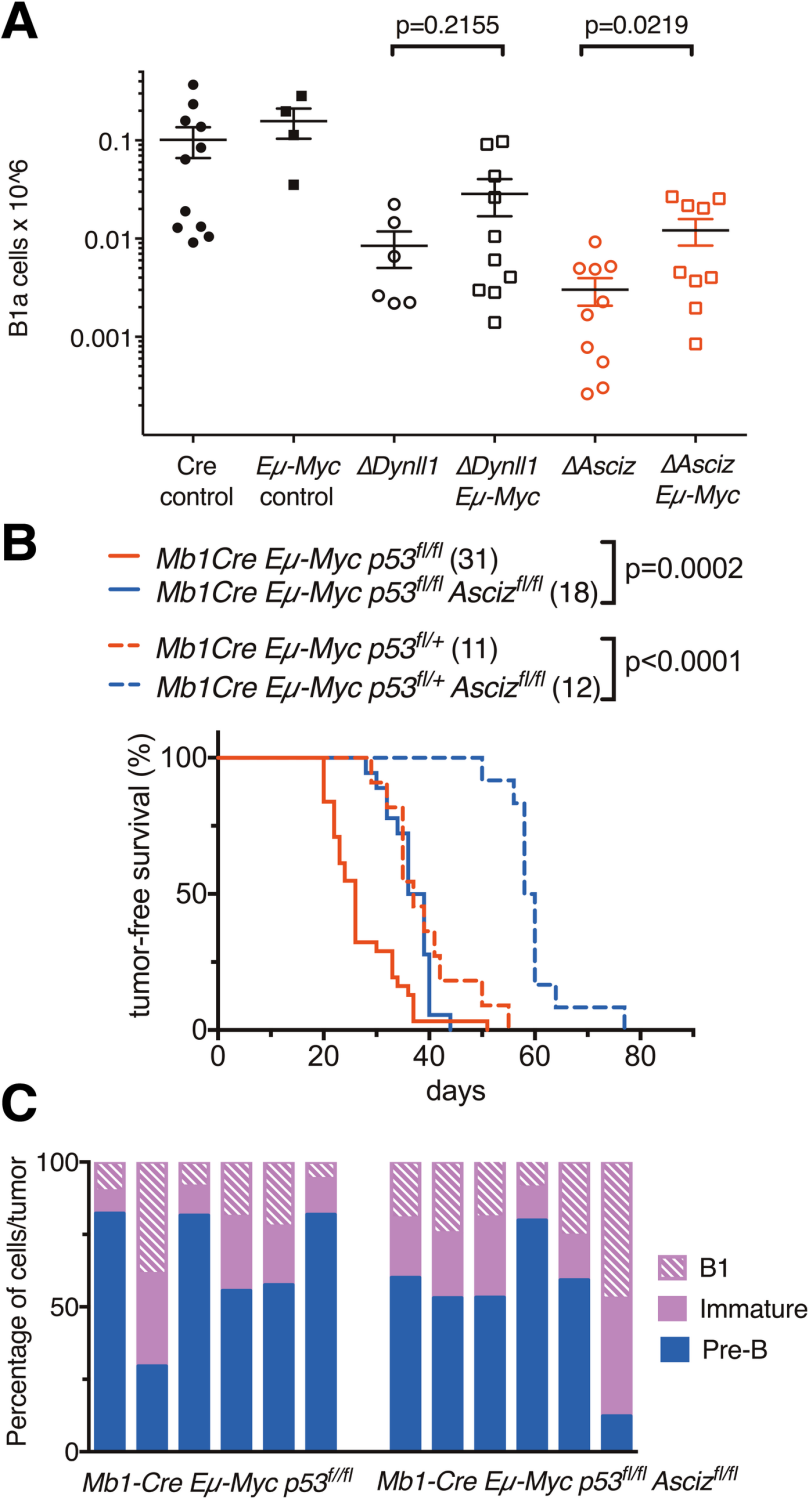
Role of the ASCIZ-DYNLL1 axis in MYC-driven B-1a-like lymphoma development. (A) Numbers of peritoneal cavity B-1a cells in 8-week old mice of the indicated genotypes (n = 4–11 in ≥3 independent experiments). Representative FACS plots of *Eμ-Myc*, *ΔDynll1 Eμ-Myc* and *ΔAsciz Eμ-Myc* mice are shown in S7 Fig. (B) Tumour-free survival of mice with the indicated genotypes. Mice were euthanised when judged to be critically ill by animal house staff who were blinded to their genotypes. (C) Distribution of B lymphoid cells in tumours of 6 randomly chosen individual mice per group. Pre-B, B220^+^ IgM^-^; immature, B220^+^ CD43^-^ IgM^+^ IgD^-^; B1, B220^+^ CD43^+^ IgM^+^ IgD^-^ CD19^+^ CD23^-^ CD11b^+^ CD5^+/-^.

We recently reported that loss of DYNLL1 (or ASCIZ) leads to a dramatic synthetic lethal interaction with oncogenic MYC expression driven by the *Eμ-Myc* transgene in B-2 lineage cells [18]. We therefore also monitored the effect of the *Eμ-Myc* transgene on B-1a cells in *Mb1-Cre Dynll1*-deleted mice. In pre-malignant 8-week-old *Eμ-Myc* mice, B-1a cell numbers in the peritoneal cavity were similar to control animals (Fig 9A and S7 Fig). Notably, B-1a cells in *Dynll1*-deleted *Eμ-Myc* mice were not further reduced relative to the low levels seen in DYNLL1-deficient mice (Fig 9A). Similar results were also observed in *Asciz*-deleted *Eμ-Myc* mice compared to *Asciz*-deleted controls (Fig 9A). In fact, in both *Asciz* or *Dynll1* mutant mice, there was a trend towards a modest rescue of the B-1a lymphopenia by *Eμ-Myc*, possibly reflecting increased MYC-driven self-renewal of the B-1a cell pool. Nevertheless, together with our previous findings, these results show that oncogenic MYC expression in combination with loss of DYNLL1 (or ASCIZ) has a specific synthetic lethal effect on the B-2 but not the B-1a cell lineage.

### The ASCIZ-DYNLL1 axis contributes to the early onset of B-1a-like lymphomas in p53-deficient *Eμ-Myc* mice

Heterozygosity of the tumour suppressor *Tp53* in *Eμ-Myc* mice leads to the extremely early development of aggressive B cell lymphomas that cause severe morbidity requiring euthanasia by 40 days of age [35]. As conventional B-2 lymphopoiesis only becomes prevalent shortly before this age, the early onset of these lymphomas in *Eμ-Myc Tp53*^*+/-*^ mice suggests that they are most likely derived from the B-1a cell lineage. To test if the ASCIZ-DYNLL1 axis contributes to the development of early postnatal lymphomas, we generated compound mutant *Mb1-Cre Tp53*^*fl*^ *Asciz*^*fl*^ *Eμ-Myc* mice. Similar to germline *Tp53*^+/-^ heterozygosity, conditional B-lymphoid-specific loss of one allele of p53 caused early-onset MYC-driven lymphomas with a median survival of 37 days (Fig 9B), compared to a median survival of ~120 days for the *Mb1-Cre Eμ-Myc* control mice on this genetic background [18]. Median survival was further reduced to 26 days in homozygous *Tp53*-deleted *Eμ-Myc* mice (Fig 9B). Importantly, conditional deletion of *Asciz* significantly extended the median survival of homozygous *Tp53*-deleted *Eμ-Myc* mice by 11 days and by 21 days in heterozygous *Tp53*-deleted *Eμ-Myc* mice. Thus, these data indicate that the ASCIZ-DYNLL1 axis is required for the rapid development and severity of these early onset B cell lymphomas in mice.

## Discussion

The data reported here demonstrate that the multi-functional dynein light chain DYNLL1 is essential for the normal development of both the adaptive and innate-like B-2 and B-1a cell lineages. Overall, the phenotypes for B cell-specific conditional *Dynll1*-deleted mice are remarkably similar to the phenotypes reported for *Asciz*-deleted mice. This congruence directly confirms the importance of DYNLL1 for B cell development in general, and also highlights DYNLL1’s role as the key effector of ASCIZ in B cells, as was previously invoked by the rescue of *Asciz*-deficiency phenotypes by retroviral complementation with DYNLL1 [17].

Our finding that ASCIZ and DYNLL1 act in a single linear pathway during the development of pre-B-2 cells (Fig 5) and B-1a cells (Fig 7A and 7B) represents a rare example where the biological functions of a transcription factor can be explained by the reduced expression of a single target gene. In the context of B cell development, this specificity is remarkable as the progression from one stage to the next is typically regulated by networks of transcription factors that activate a wide range of functionally important effectors [36, 37]. However, the synergistic interaction between loss of *Asciz* and loss of *Dynll1* at the immature B cell stage implies that these proteins must also have some non-overlapping, developmental stage-specific functions. Moreover, this synergistic effect also shows that—on an *Asciz*-deficient background—very low residual levels of DYNLL1 can still support some molecular functions that are lost in the complete absence of the protein in *Asciz/Dynll1* double mutant animals.

An important mechanistic finding was that the defects in B-2 lineage development caused by the absence of DYNLL1 could be rescued by deletion of *Bim*, but not deletion of *tp53*, and that they were associated with significantly reduced levels of BCL-2. The p53-independence of the G1-phase delay seen in *Dynll1*-deleted cycling pre-B cells indicates that this delay is not due to a primary cell cycle defect or unrepaired DNA lesions. Cell cycle entry at the fraction C' stage of B cell development depends on mitogenic signals from stromal cell derived cytokines, such as IL-7, and tonic ligand-independent activity of the pre-BCR [38, 39]. Interestingly, impaired IL-7 signalling is known to diminish cell proliferation at the pre-B cell stage and also causes apoptosis at this stage of B cell development and also at the immature B cell stage [29, 30]. IL-7 is required for high-level BCL-2 expression [40], and B cell development defects in IL7- or IL-7R deficient mice can be suppressed by the loss of BIM [29, 30]. Based on these similarities between the phenotypes of these animals and those of the *Mb1-Cre Dynll1*-deleted mice (Figs 3 and 4), a plausible explanation for our observations is that DYNLL1 may be playing an important role in the IL-7/IL-7R signaling pathway in developing B lymphoid cells. Likewise, as BCR activation in mature B cells, which utilizes similar signal transductions pathways to the pre-BCR, has recently been shown to be enhanced by cytoplasmic dynein-1-mediated receptor clustering [41], it would be conceivable that DYNLL1 could stimulate pre-BCR signaling through a similar dynein-mediated process. Finally, another relevant DYNLL1 effector in this setting could the human-immunodeficiency-related guanine-nucleotide-exchange factor RasGRP1 [42], which is known to be particularly critical for B-1a cell development in mice [43].

A notable difference between the phenotypes of *Mb1-Cre Dynll1*-deleted and *Mb1-Cre Asciz*-deleted mice was that the pre-arranged *SW*_*HEL*_ BCR transgene could partially rescue immature B cell numbers in the absence of DYNLL1, in contrast to having no effect on ASCIZ-deficient cells [17]. A possible explanation for this difference could be that low levels of DYNLL1 may be necessary and sufficient for V(D)J recombination of the *Ig* gene loci. As our epistasis analysis indicates that *Asciz* and *Dynll1* function in a linear pathway during early pro-B/pre-B cell development, including during the stage when the *Igh* locus gets rearranged, but have partially divergent functions during later developmental stages (Fig 5A), this suggests that the effect of DYNLL1 would be specific to the recombination of the *Igλ/κ* light chain loci. In this context it is interesting to note that DYNLL1 preferentially binds to TQT sequence motifs, which are also potential phosphorylation site motifs for the family of ATM-related kinases [44], including the DNA-dependent protein kinase that is critical for V(D)J recombination [45] and the repair scaffold protein 53BP1 [46].

Within the B-2 cell lineage, it may seem somewhat paradoxical that loss of DYNLL1 only affected the number of follicular B cells but not the number of marginal zone B cells (S1 Fig), even though both are generated by the same B cell development pathway in the bone marrow. This finding is reminiscent of other mouse models with severely reduced follicular B cell numbers but normal marginal zone B cell numbers, for example mice lacking the BAF chromatin remodelling complex subunit BAF155 [47], or the phosphatidylserine translocase ATP11C [48, 49]. As indicated above, marginal zone B cells have a much longer lifespan than follicular B cells [27], and they are also present at a much lower number (S1 Fig). So, it is plausible that the marginal zone compartment reaches a steady state faster than the more ephemeral follicular compartment.

A major finding of this study is that loss of DYNLL1 or ASCIZ had even more severe quantitative effects on the development of the innate-like B-1a cell lineage (~100-fold reductions in initial B-1a cell pools; Fig 7B) than on B-2 cells (~10-fold reductions of mature splenic B cells; Fig 1D). In both B-2 (Fig 2) and B-1a cell (Fig 7) development, loss of DYNLL1 leads to significant cell losses at the pre-B stages, which become more dramatic during the immature/transitional stages when B-2 and B-1a lineage cells first become surface-IgM positive. It is thus notable that the loss of *Bim* and expression of the *SW*_*HEL*_ BCR transgene, which can overcome developmental defects caused by loss of DYNLL1 during this stage in the B-2 cell lineage, did not have a comparable rescue effect for B-1a cells. This differential effect of the *SW*_*HEL*_ transgene, which even worsened the B-1a lymphopenia in *Dynll1*-deleted mice (Fig 8), is best explained by the concept that B-1a and B-2 cells have distinctive Ig repertoires. Selection of B-1a lineage-biased IgH chains seems to involve their relatively poor association with the surrogate light chains, and weak antigen affinity of their ultimate BCRs [2]. In contrast, the *SW*_*HEL*_ BCR is specific to B-2 lymphocytes, and even B-1a cells of *V*_*H*_*10* heavy chain heterozygous *SW*_*HEL*_ mice do not recognize the HEL antigen [33]. This explains why the homozygous *V*_*H*_*10* heavy chain knock-in (which restricts its allelic exclusion during *Igh* VDJ recombination in pro-B cells) in our *SW*_*HEL*_ mice, even without the *V*_*κ*_*10* light chain transgene, led to a notable reduction of B-1a cells, and a corresponding compounding effect on the B-1a lymphopenia in *Dynll1* mutant animals (Fig 8B). It would be interesting to test in the future if a transgenic B-1a cell-specific BCR transgene—for example, the phosphatidylcholine-specific V_H_12/V_κ6_BCR—could suppress the B-1a cell developmental defect of *Dynll1*-deleted mice, similar to the partial rescue of their B-2 cell defect by the *SW*_*HEL*_ transgene.

The pro-apoptotic BH3-only protein BIM has a well-documented critical function in B-2 development to eliminate immature B cells expressing auto-reactive BCRs [50]. In contrast, B-1a cells appear to be selected, at least in part, towards weak auto-reactive BCR specificities [2]. So, it makes sense that BCR ligation by self-antigens does not activate apoptotic cell death programs in this lineage. This is also consistent with the notion that survival of B-1a cells, in marked contrast to B-2 cells, does not depend on the BAFF-R/NFκB pathway, one of the key effector functions of which is to upregulate anti-apoptotic BCL-2 family proteins, such as BCL-2 and MCL-1, which both inhibit BIM-driven apoptosis [51].

B-1a cells have attracted renewed attention based on their widely appreciated roles in innate immune signaling [52, 53] and their role in the origin of subsets of both acute lymphoblastic and chronic lymphocytic leukemias [54, 55]. It has become clear that functional and developmental differences between B-1a and B-2 cells are driven by divergent transcriptional programs, growth factor sensitivities, and BCR repertoires. Based on the differential roles of *Dynll1* in B-2 versus B-1a cells, our findings here add *Bim* and *Myc* to the list of genetic factors with highly distinctive lineage-specific functions in B cell development.

## Materials and methods

### Ethics statement

Animal experiments were performed according to the Australian Code for the Care and Use of Animals for Scientific Purposes, 8th Edition (2013), and approved by the St. Vincent’s Hospital Melbourne Animal Ethics Committee, approval numbers 047/12, 019/13, and 002/17.

### Mice

Mice were housed in specific pathogen-free micro-isolators. All mouse mutations were generated on an inbred C57BL/6 background, or had been backcrossed to the C57BL/6 background for at least 10 generations. Conditional *Asciz [56]*, *Bim* [57], *Dynll1* [18] and *tp53* [58] alleles, *Bim* knockout [31], *Mb1-Cre [26]*, *SW*_*HEL* [__33__]_ and *Eμ-Myc* [59] mice have been described before. *SW*_*HEL*_ mice used here were homozygous for the anti-HEL *V*_*H*_*10* heavy-chain knock-in, and contained an anti-HEL *Vκ10* light chain transgene. Primers used for genotyping are listed in references [17, 18].

Mice were analysed at the ages indicated in the figure legends. Ethical endpoints of survival analyses of tumor-prone mice were determined by trained animal technician who were blinded to the genotypes. Mice with different genotypes were co-housed in the same micro-isolators.

### Flow cytometry

Spleen, fetal liver, bone marrow and tumour cell suspensions were prepared as described [18]. Bone marrow cellularities included tibias and femurs of two legs. Peritoneal B-1a cells were isolated by lavage using a syringe connected to 26G and then 23G needles with PBS containing 2% FBS. Cell counts were determined using an automated cell counter (KX-21N, Sysmex). Stained cells were analyzed using an LSRFortessa (BD) using FACSDiva (BD) and FlowJo (Tree Star) software. Our gating strategy for analysis of bone marrow cells is shown in S3A Fig of reference [18]. Mature splenic B cells were isolated using the MACS mouse B cell isolation kit (Miltenyi Biotec). FACS sorting of bone marrow B cells was performed using a FACSAria (BD). IgM-depleted bone marrow pre-B cells were isolated using MACS Streptavidin MicroBeads (Miltenyi Biotec). Leukocytes were then incubated overnight in DMEM with 10% FBS at 37°C to enhance pre-BCR expression before staining and FACS analysis.

The following reagents were used for staining: B220-APC (eBioscience), B220-FITC (Biolegend), BP1-PE (BD), CD1d FITC (BD), CD3ε-biotin (eBioscience), CD5-FITC (BD), CD8α-biotin (Biolegend), CD11b-APC-Cy7 (BD), CD11b-biotin (Biolegend), CD19-APC eFluor780 (eBioscience), CD19-PerCP-Cy5.5 (eBioscience), CD21-PE (Biolegend), CD21/35 APC (BD), CD23-biotin (BD), CD24-FITC (BD), CD43-PE (BD), CD93-PerCP-Cy5.5 (eBioscience), GR1-biotin (eBioscience), IgD-eFluor450 (eBioscience), IgM-biotin (eBioscience), IgM-PE-Cy7 (BD), IgM(b) Biotin (BD), NK1.1-biotin (Biolegend), Pre-B Cell Receptor Biotin (SL156, BD), Streptavidin-BV605 (Biolegend), Ter119-biotin (eBioscience) and AnnexinV-eFluor450 detection kit (eBioscience).

### Immunoblots and quantitative PCR

Western blot analysis was performed as described [18] using antibodies against Actin (EMD Millipore/Merck, MAB1501; loading control), BAK (Sigma, B5897), BAX (WEHI, 49F9-13-3; obtained from David Huang), BCL-2 (Cell Signaling Technology, 50E3) BCL-XL (BD, 610212), BIM (Enzo, 3C5), DYNLL1 (Abcam, ab51603), MCL-1 (Cell Signaling, D35A5), M601 (Abcam, ab110411), tubulin (Sigma, T9026), and XRCC1 (Santa Cruz, sc-11429), horseradish peroxidase-coupled secondary antibodies and ECL reagents (GE Healthcare) antibodies. Western blots were quantified as described [60]. Subcellular fractionation assays were performed using a cell fractionation kit (Abcam, ab109719).

Total RNA was isolated using Isolate II RNA Micro Kit (Bioline) and reverse transcribed using a High Capacity cDNA Reverse Transcription kit (Applied Biosystems) following the manufacturer’s protocol. Real time PCR was performed using AmpliTaq Gold DNA Polymerase (Applied Biosystems) and a LightCycler 480 (Roche). TaqMan primers for *Bcl2* and *Hmbs* were purchased from ThermoFisher Scientific. Ct values were normalized to *Hmbs* as the endogenous control and expressed as deltaCt (dCT).

### Statistical analysis

Statistical analysis was performed using GraphPad Prism software (San Diego). P values were calculated by two-tailed unpaired Student’s t test, or Mantel-Cox test for survival analyses. Grouped data were analyzed using the multiple T test function (one upaired t test per row, each row analysed individually) using the two-stage linear step-up procedure of Benjamini, Krieger and Yekutieli. Error bars represent the mean ± SEM. Numbers of mice per analysis, and numbers of independent experiments are indicated in the figures or legends.

## Supporting information

S1 Fig Normal numbers of marginal zone B cells in *Dynll1*-deleted mice. (A) Representative FACS plots and gating strategies for the analysis of follicular and marginal zone B cells. (B, C) Quantification (mean ± S.E.) of follicular B cells (B) and marginal zone B cells (C) in the spleens of 8-10-week old mice.
(TIF)

S2 Fig FACS analysis of fraction D-E cells. (A) FACS plots of B220^+^CD43^-^ bone marrow cells from one *Mb1-Cre* control mouse, one *Dynll1*^*fl/fl*^ control mouse and two *Dynll1*-deleted mice with fractions D (small pre-B), E (immature) and F (recirclating) highlighted. (B) Mean fluorescence intensity (M.F.I.) of the IgM signal in fraction E cells (mean ± S.E.).
(TIF)

S3 Fig Pre-BCR and BCR expression is not affected in *Dynll1*-deleted B-cells. (A, B) Representative FACS plots and gating strategies to determine pre-BCR (SL156) expression on bone marrow pre-B lymphocytes (mean ± S.E.) in 8-to-10-week old mice. (C) Mean fluorescence intensity (M.F.I.) of the pre-BCR (SL156) signal on CD43^+^B220^+^ bone marrow lymphocytes (mean ± S.E.).
(TIF)

S4 Fig Comparison of B-2 cell developmental defects in *Dynll1*-deleted, *Asciz*-deleted and *Asciz/Dynll1*-double deleted mice. Representative FACS plots indicate gating strategies to determine total and mature splenic B cell numbers.
(TIF)

S5 Fig Peritoneal cavity B-1a cell analysis of 8-week old mice. Representative FACS plots used for the quantification of B-1a cell numbers in the peritoneal cavity of 8-week old mice in Fig 7.
(TIF)

S6 Fig Effects of *Bim* knockout and *SW*_*HEL*_ complementation on the B-1a lymphopenia in *Dynll1*-deleted mice. Representative FACS plots of peritoneal cavity cells of 8-week old mice related to the quantitative analyses in Fig 8.
(TIF)

S7 Fig Effect of oncogenic MYC expression on B-1a cells. Representative FACS plots of peritoneal cavity cells of 8-week old mice, related to Fig 9A.
(TIF)

## References

[1] LeBien TW, Tedder TF. B lymphocytes: how they develop and function. Blood. 2008;112(5):1570–80. Epub 2008/08/30. doi: 10.1182/blood-2008-02-078071 18725575 10.1182/blood-2008-02-078071 PMC2518873

[2] Hardy RR. B-1 B cell development. J Immunol. 2006;177(5):2749–54. 16920907 10.4049/jimmunol.177.5.2749

[3] Montecino-Rodriguez E, Dorshkind K. B-1 B cell development in the fetus and adult. Immunity. 2012;36(1):13–21. doi: 10.1016/j.immuni.2011.11.017 22284417 10.1016/j.immuni.2011.11.017 PMC3269035

[4] Melchers F. Checkpoints that control B cell development. J Clin Invest. 2015;125(6):2203–10. doi: 10.1172/JCI78083 25938781 10.1172/JCI78083 PMC4497745

[5] Hardy RR, Carmack CE, Shinton SA, Kemp JD, Hayakawa K. Resolution and characterization of pro-B and pre-pro-B cell stages in normal mouse bone marrow. J Exp Med. 1991;173(5):1213–25. Epub 1991/05/01. 1827140 10.1084/jem.173.5.1213 PMC2118850

[6] Enders A, Bouillet P, Puthalakath H, Xu Y, Tarlinton DM, Strasser A. Loss of the pro-apoptotic BH3-only Bcl-2 family member Bim inhibits BCR stimulation-induced apoptosis and deletion of autoreactive B cells. J Exp Med. 2003;198(7):1119–26. Epub 2003/10/01. doi: 10.1084/jem.20030411 [pii]. 14517273 10.1084/jem.20030411 PMC2194219

[7] Ghosn EE, Yang Y. Hematopoietic stem cell-independent B-1a lineage. Ann N Y Acad Sci. 2015;1362:23–38. doi: 10.1111/nyas.12881 26662720 10.1111/nyas.12881

[8] Montecino-Rodriguez E, Dorshkind K. Formation of B-1 B cells from neonatal B-1 transitional cells exhibits NF-kappaB redundancy. J Immunol. 2011;187(11):5712–9. doi: 10.4049/jimmunol.1102416 22031760 10.4049/jimmunol.1102416 PMC3221773

[9] Pedersen GK, Adori M, Khoenkhoen S, Dosenovic P, Beutler B, Karlsson Hedestam GB. B-1a transitional cells are phenotypically distinct and are lacking in mice deficient in IkappaBNS. Proc Natl Acad Sci U S A. 2014;111(39):E4119–26. doi: 10.1073/pnas.1415866111 25228759 10.1073/pnas.1415866111 PMC4191775

[10] Yang Y, Wang C, Yang Q, Kantor AB, Chu H, Ghosn EE, et al Distinct mechanisms define murine B cell lineage immunoglobulin heavy chain (IgH) repertoires. Elife. 2015;4:e09083 doi: 10.7554/eLife.09083 26422511 10.7554/eLife.09083 PMC4714975

[11] Montecino-Rodriguez E, Fice M, Casero D, Berent-Maoz B, Barber CL, Dorshkind K. Distinct Genetic Networks Orchestrate the Emergence of Specific Waves of Fetal and Adult B-1 and B-2 Development. Immunity. 2016;45:527–39. doi: 10.1016/j.immuni.2016.07.012 27566938 10.1016/j.immuni.2016.07.012 PMC5033716

[12] McNees CJ, Conlan LA, Tenis N, Heierhorst J. ASCIZ regulates lesion-specific Rad51 focus formation and apoptosis after methylating DNA damage. EMBO J. 2005;24(13):2447–57. doi: 10.1038/sj.emboj.7600704 15933716 10.1038/sj.emboj.7600704 PMC1173145

[13] Loizou JI, Sancho R, Kanu N, Bolland DJ, Yang F, Rada C, et al ATMIN Is Required for Maintenance of Genomic Stability and Suppression of B Cell Lymphoma. Cancer Cell. 2011;19(5):587–600. Epub 2011/05/18. doi: 10.1016/j.ccr.2011.03.022 21575860 10.1016/j.ccr.2011.03.022 PMC4452547

[14] Jurado S, Conlan LA, Baker EK, Ng JL, Tenis N, Hoch NC, et al ATM substrate Chk2-interacting Zn2+ finger (ASCIZ) Is a bi-functional transcriptional activator and feedback sensor in the regulation of dynein light chain (DYNLL1) expression. J Biol Chem. 2012;287(5):3156–64. Epub 2011/12/15. doi: 10.1074/jbc.M111.306019 22167198 10.1074/jbc.M111.306019 PMC3270970

[15] Zaytseva O, Tenis N, Mitchell N, Kanno S, Yasui A, Heierhorst J, et al The Novel Zinc Finger Protein dASCIZ Regulates Mitosis in Drosophila via an Essential Role in Dynein Light-Chain Expression. Genetics. 2014;196(2):443–53. Epub 2013/12/18. doi: 10.1534/genetics.113.159541 24336747 10.1534/genetics.113.159541 PMC3914618

[16] Goggolidou P, Stevens JL, Agueci F, Keynton J, Wheway G, Grimes DT, et al ATMIN is a transcriptional regulator of both lung morphogenesis and ciliogenesis. Development. 2014;141(20):3966–77. Epub 2014/10/09. doi: 10.1242/dev.107755 25294941 10.1242/dev.107755 PMC4197704

[17] Jurado S, Gleeson K, O'Donnell K, Izon DJ, Walkley CR, Strasser A, et al The Zinc-finger protein ASCIZ regulates B cell development via DYNLL1 and Bim. J Exp Med. 2012;209(9):1629–39. Epub 2012/08/15. doi: 10.1084/jem.20120785 22891272 10.1084/jem.20120785 PMC3428950

[18] Wong DM, Li L, Jurado S, King A, Bamford R, Wall M, et al The transcription factor ASCIZ and its target DYNLL1 are essential for the development and expansion of MYC-driven B cell lymphoma. Cell Rep. 2016;14:1488–99. doi: 10.1016/j.celrep.2016.01.012 26832406 10.1016/j.celrep.2016.01.012

[19] King SM, Patel-King RS. The M(r) = 8,000 and 11,000 outer arm dynein light chains from Chlamydomonas flagella have cytoplasmic homologues. J Biol Chem. 1995;270(19):11445–52. Epub 1995/05/12. 7744782 10.1074/jbc.270.19.11445

[20] Williams JC, Roulhac PL, Roy AG, Vallee RB, Fitzgerald MC, Hendrickson WA. Structural and thermodynamic characterization of a cytoplasmic dynein light chain-intermediate chain complex. Proc Natl Acad Sci U S A. 2007;104(24):10028–33. Epub 2007/06/07. doi: 10.1073/pnas.0703614104 17551010 10.1073/pnas.0703614104 PMC1885999

[21] Nyarko A, Barbar E. Light chain-dependent self-association of dynein intermediate chain. J Biol Chem. 2011;286(2):1556–66. Epub 2010/10/27. doi: 10.1074/jbc.M110.171686 20974845 10.1074/jbc.M110.171686 PMC3020764

[22] Asante D, Stevenson NL, Stephens DJ. Subunit composition of the human cytoplasmic dynein-2 complex. J Cell Sci. 2014;127(Pt 21):4774–87. doi: 10.1242/jcs.159038 25205765 10.1242/jcs.159038 PMC4215718

[23] Barbar E. Dynein light chain LC8 is a dimerization hub essential in diverse protein networks. Biochemistry. 2008;47(2):503–8. Epub 2007/12/21. doi: 10.1021/bi701995m 18092820 10.1021/bi701995m

[24] Rapali P, Szenes A, Radnai L, Bakos A, Pal G, Nyitray L. DYNLL/LC8: a light chain subunit of the dynein motor complex and beyond. FEBS J. 2011;278(17):2980–96. Epub 2011/07/23. doi: 10.1111/j.1742-4658.2011.08254.x 21777386 10.1111/j.1742-4658.2011.08254.x

[25] Puthalakath H, Huang DC, O'Reilly LA, King SM, Strasser A. The proapoptotic activity of the Bcl-2 family member Bim is regulated by interaction with the dynein motor complex. Mol Cell. 1999;3(3):287–96. Epub 1999/04/13. 10198631 10.1016/s1097-2765(00)80456-6

[26] Hobeika E, Thiemann S, Storch B, Jumaa H, Nielsen PJ, Pelanda R, et al Testing gene function early in the B cell lineage in mb1-cre mice. Proc Natl Acad Sci U S A. 2006;103(37):13789–94. Epub 2006/08/31. doi: 10.1073/pnas.0605944103 16940357 10.1073/pnas.0605944103 PMC1564216

[27] Hao Z, Rajewsky K. Homeostasis of peripheral B cells in the absence of B cell influx from the bone marrow. J Exp Med. 2001;194(8):1151–64. 11602643 10.1084/jem.194.8.1151 PMC2193512

[28] McKinley KL, Cheeseman IM. Large-Scale Analysis of CRISPR/Cas9 Cell-Cycle Knockouts Reveals the Diversity of p53-Dependent Responses to Cell-Cycle Defects. Dev Cell. 2017;40(4):405–20 e2. doi: 10.1016/j.devcel.2017.01.012 28216383 10.1016/j.devcel.2017.01.012 PMC5345124

[29] Oliver PM, Wang M, Zhu Y, White J, Kappler J, Marrack P. Loss of Bim allows precursor B cell survival but not precursor B cell differentiation in the absence of interleukin 7. J Exp Med. 2004;200(9):1179–87. Epub 2004/11/03. doi: 10.1084/jem.20041129 15520248 10.1084/jem.20041129 PMC2211863

[30] Huntington ND, Labi V, Cumano A, Vieira P, Strasser A, Villunger A, et al Loss of the pro-apoptotic BH3-only Bcl-2 family member Bim sustains B lymphopoiesis in the absence of IL-7. Int Immunol. 2009;21(6):715–25. doi: 10.1093/intimm/dxp043 19454543 10.1093/intimm/dxp043 PMC2980998

[31] Bouillet P, Metcalf D, Huang DC, Tarlinton DM, Kay TW, Kontgen F, et al Proapoptotic Bcl-2 relative Bim required for certain apoptotic responses, leukocyte homeostasis, and to preclude autoimmunity. Science. 1999;286(5445):1735–8. Epub 1999/11/27 10576740 10.1126/science.286.5445.1735

[32] Anjos-Afonso F, Loizou JI, Bradburn A, Kanu N, Purewal S, Da Costa C, et al Perturbed hematopoiesis in mice lacking ATMIN. Blood. 2016;128:2017–21. doi: 10.1182/blood-2015-09-672980 27581360 10.1182/blood-2015-09-672980 PMC5147016

[33] Phan TG, Amesbury M, Gardam S, Crosbie J, Hasbold J, Hodgkin PD, et al B cell receptor-independent stimuli trigger immunoglobulin (Ig) class switch recombination and production of IgG autoantibodies by anergic self-reactive B cells. J Exp Med. 2003;197(7):845–60. Epub 2003/04/02. doi: 10.1084/jem.20022144 [pii]. 12668643 10.1084/jem.20022144 PMC2193892

[34] Montecino-Rodriguez E, Leathers H, Dorshkind K. Identification of a B-1 B cell-specified progenitor. Nat Immunol. 2006;7(3):293–301. doi: 10.1038/ni1301 16429139 10.1038/ni1301

[35] Michalak EM, Jansen ES, Happo L, Cragg MS, Tai L, Smyth GK, et al Puma and to a lesser extent Noxa are suppressors of Myc-induced lymphomagenesis. Cell Death Differ. 2009;16(5):684–96. Epub 2009/01/17. doi: 10.1038/cdd.2008.195 19148184 10.1038/cdd.2008.195 PMC2743939

[36] Busslinger M. Transcriptional control of early B cell development. Annu Rev Immunol. 2004;22:55–79. Epub 2004/03/23. doi: 10.1146/annurev.immunol.22.012703.104807 15032574 10.1146/annurev.immunol.22.012703.104807

[37] Nutt SL, Kee BL. The transcriptional regulation of B cell lineage commitment. Immunity. 2007;26(6):715–25. Epub 2007/06/22. doi: 10.1016/j.immuni.2007.05.010 17582344 10.1016/j.immuni.2007.05.010

[38] Herzog S, Reth M, Jumaa H. Regulation of B-cell proliferation and differentiation by pre-B-cell receptor signalling. Nat Rev Immunol. 2009;9(3):195–205. Epub 2009/02/26. doi: 10.1038/nri2491 19240758 10.1038/nri2491

[39] Saijo K, Schmedt C, Su IH, Karasuyama H, Lowell CA, Reth M, et al Essential role of Src-family protein tyrosine kinases in NF-kappaB activation during B cell development. Nat Immunol. 2003;4(3):274–9. Epub 2003/02/04. doi: 10.1038/ni893 [pii]. 12563261 10.1038/ni893

[40] Lu L, Chaudhury P, Osmond DG. Regulation of cell survival during B lymphopoiesis: apoptosis and Bcl-2/Bax content of precursor B cells in bone marrow of mice with altered expression of IL-7 and recombinase-activating gene-2. J Immunol. 1999;162(4):1931–40. 9973461

[41] Schnyder T, Castello A, Feest C, Harwood NE, Oellerich T, Urlaub H, et al B cell receptor-mediated antigen gathering requires ubiquitin ligase Cbl and adaptors Grb2 and Dok-3 to recruit dynein to the signaling microcluster. Immunity. 2011;34(6):905–18. Epub 2011/06/28. doi: 10.1016/j.immuni.2011.06.001 21703542 10.1016/j.immuni.2011.06.001

[42] Salzer E, Cagdas D, Hons M, Mace EM, Garncarz W, Petronczki OY, et al RASGRP1 deficiency causes immunodeficiency with impaired cytoskeletal dynamics. Nat Immunol. 2016;17(12):1352–60. doi: 10.1038/ni.3575 27776107 10.1038/ni.3575 PMC6400263

[43] Guo B, Rothstein TL. RasGRP1 Is an Essential Signaling Molecule for Development of B1a Cells with Autoantigen Receptors. J Immunol. 2016;196(6):2583–90. doi: 10.4049/jimmunol.1502132 26851222 10.4049/jimmunol.1502132 PMC5548536

[44] Traven A, Heierhorst J. SQ/TQ cluster domains: concentrated ATM/ATR kinase phosphorylation site regions in DNA-damage-response proteins. Bioessays. 2005;27(4):397–407. Epub 2005/03/17. doi: 10.1002/bies.20204 15770685 10.1002/bies.20204

[45] Oksenych V, Kumar V, Liu X, Guo C, Schwer B, Zha S, et al Functional redundancy between the XLF and DNA-PKcs DNA repair factors in V(D)J recombination and nonhomologous DNA end joining. Proc Natl Acad Sci U S A. 2013;110(6):2234–9. Epub 2013/01/25. doi: 10.1073/pnas.1222573110 23345432 10.1073/pnas.1222573110 PMC3568359

[46] Lo KW, Kan HM, Chan LN, Xu WG, Wang KP, Wu Z, et al The 8-kDa dynein light chain binds to p53-binding protein 1 and mediates DNA damage-induced p53 nuclear accumulation. J Biol Chem. 2005;280(9):8172–9. Epub 2004/12/22. doi: 10.1074/jbc.M411408200 15611139 10.1074/jbc.M411408200

[47] Choi J, Ko M, Jeon S, Jeon Y, Park K, Lee C, et al The SWI/SNF-like BAF complex is essential for early B cell development. J Immunol. 2012;188(8):3791–803. doi: 10.4049/jimmunol.1103390 22427636 10.4049/jimmunol.1103390

[48] Siggs OM, Arnold CN, Huber C, Pirie E, Xia Y, Lin P, et al The P4-type ATPase ATP11C is essential for B lymphopoiesis in adult bone marrow. Nat Immunol. 2011;12(5):434–40. doi: 10.1038/ni.2012 21423172 10.1038/ni.2012 PMC3079768

[49] Yabas M, Teh CE, Frankenreiter S, Lal D, Roots CM, Whittle B, et al ATP11C is critical for the internalization of phosphatidylserine and differentiation of B lymphocytes. Nat Immunol. 2011;12(5):441–9. doi: 10.1038/ni.2011 21423173 10.1038/ni.2011 PMC3272780

[50] Strasser A. The role of BH3-only proteins in the immune system. Nat Rev Immunol. 2005;5(3):189–200. Epub 2005/02/19. doi: 10.1038/nri1568 15719025 10.1038/nri1568

[51] Vincent FB, Saulep-Easton D, Figgett WA, Fairfax KA, Mackay F. The BAFF/APRIL system: emerging functions beyond B cell biology and autoimmunity. Cytokine Growth Factor Rev. 2013;24(3):203–15. doi: 10.1016/j.cytogfr.2013.04.003 23684423 10.1016/j.cytogfr.2013.04.003 PMC7108297

[52] Beaudin AE, Forsberg EC. To B1a or not to B1a: do hematopoietic stem cells contribute to tissue-resident immune cells? Blood. 2016;128:2765–9. doi: 10.1182/blood-2016-10-697813 27799163 10.1182/blood-2016-10-697813 PMC5159701

[53] Rothenberg EV. Multiple Curricula for B Cell Developmental Programming. Immunity. 2016;45(3):457–8. doi: 10.1016/j.immuni.2016.09.005 27653594 10.1016/j.immuni.2016.09.005

[54] Montecino-Rodriguez E, Li K, Fice M, Dorshkind K. Murine B-1 B cell progenitors initiate B-acute lymphoblastic leukemia with features of high-risk disease. J Immunol. 2014;192(11):5171–8. doi: 10.4049/jimmunol.1303170 24752443 10.4049/jimmunol.1303170 PMC4028370

[55] Hayakawa K, Formica AM, Brill-Dashoff J, Shinton SA, Ichikawa D, Zhou Y, et al Early generated B1 B cells with restricted BCRs become chronic lymphocytic leukemia with continued c-Myc and low Bmf expression. J Exp Med. 2016;213:3007–24. doi: 10.1084/jem.20160712 27899442 10.1084/jem.20160712 PMC5154941

[56] Jurado S, Smyth I, van Denderen B, Tenis N, Hammet A, Hewitt K, et al Dual functions of ASCIZ in the DNA base damage response and pulmonary organogenesis. PLoS Genet. 2010;6(10):e1001170 Epub 2010/10/27. doi: 10.1371/journal.pgen.1001170 20975950 10.1371/journal.pgen.1001170 PMC2958817

[57] Herold MJ, Stuchbery R, Merino D, Willson T, Strasser A, Hildeman D, et al Impact of conditional deletion of the pro-apoptotic BCL-2 family member BIM in mice. Cell Death Dis. 2014;5:e1446 doi: 10.1038/cddis.2014.409 25299771 10.1038/cddis.2014.409 PMC4237241

[58] Jonkers J, Meuwissen R, van der Gulden H, Peterse H, van der Valk M, Berns A. Synergistic tumor suppressor activity of BRCA2 and p53 in a conditional mouse model for breast cancer. Nat Genet. 2001;29(4):418–25. Epub 2001/11/06. doi: 10.1038/ng747 [pii]. 11694875 10.1038/ng747

[59] Adams JM, Harris AW, Pinkert CA, Corcoran LM, Alexander WS, Cory S, et al The c-myc oncogene driven by immunoglobulin enhancers induces lymphoid malignancy in transgenic mice. Nature. 1985;318(6046):533–8. 3906410 10.1038/318533a0

[60] Liu R, King A, Hoch NC, Chang C, Kelly GL, Deans AJ, et al ASCIZ/ATMIN is dispensable for ATM signaling in response to replication stress. DNA Repair (Amst). 2017;57:29–34. doi: 10.1016/j.dnarep.2017.06.022 28648892 10.1016/j.dnarep.2017.06.022 PMC5576915

