# Supplementary figures and images for: Dynein light chain regulates adaptive and innate B cell development by distinctive genetic mechanisms
Source: PLoS Genet. 2017 Sep 18;13(9):e1007010. doi: 10.1371/journal.pgen.1007010 (PMC5619840; doi:10.1371/journal.pgen.1007010)

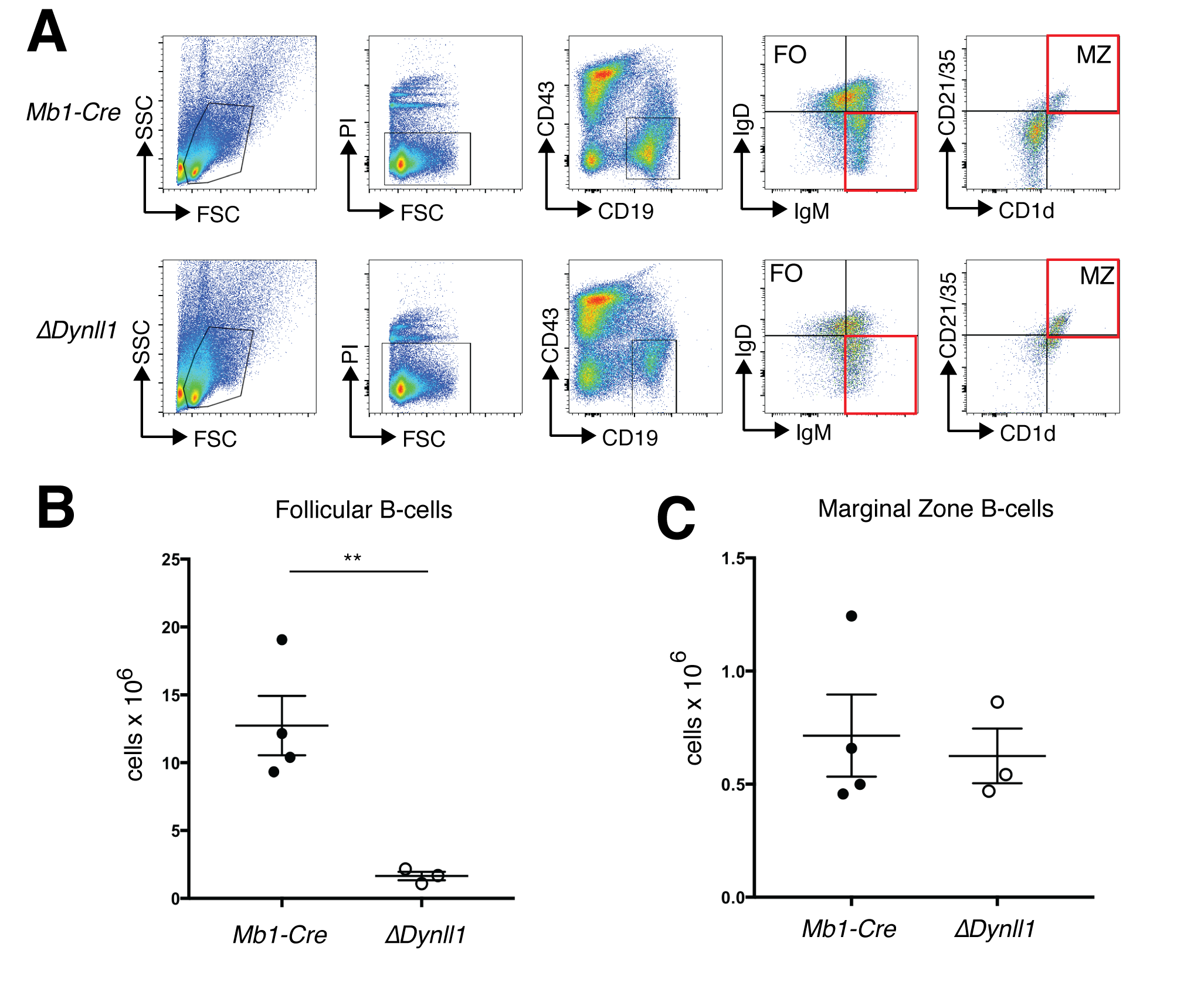

Supplement: S1 Fig — (A) Representative FACS plots and gating strategies for the analysis of follicular and marginal zone B cells. (B, C) Quantification (mean ± S.E.) of follicular B cells (B) and marginal zone B cells (C) in the spleens of 8-10-week old mice. (TIF) [file pgen.1007010.s001.tif]

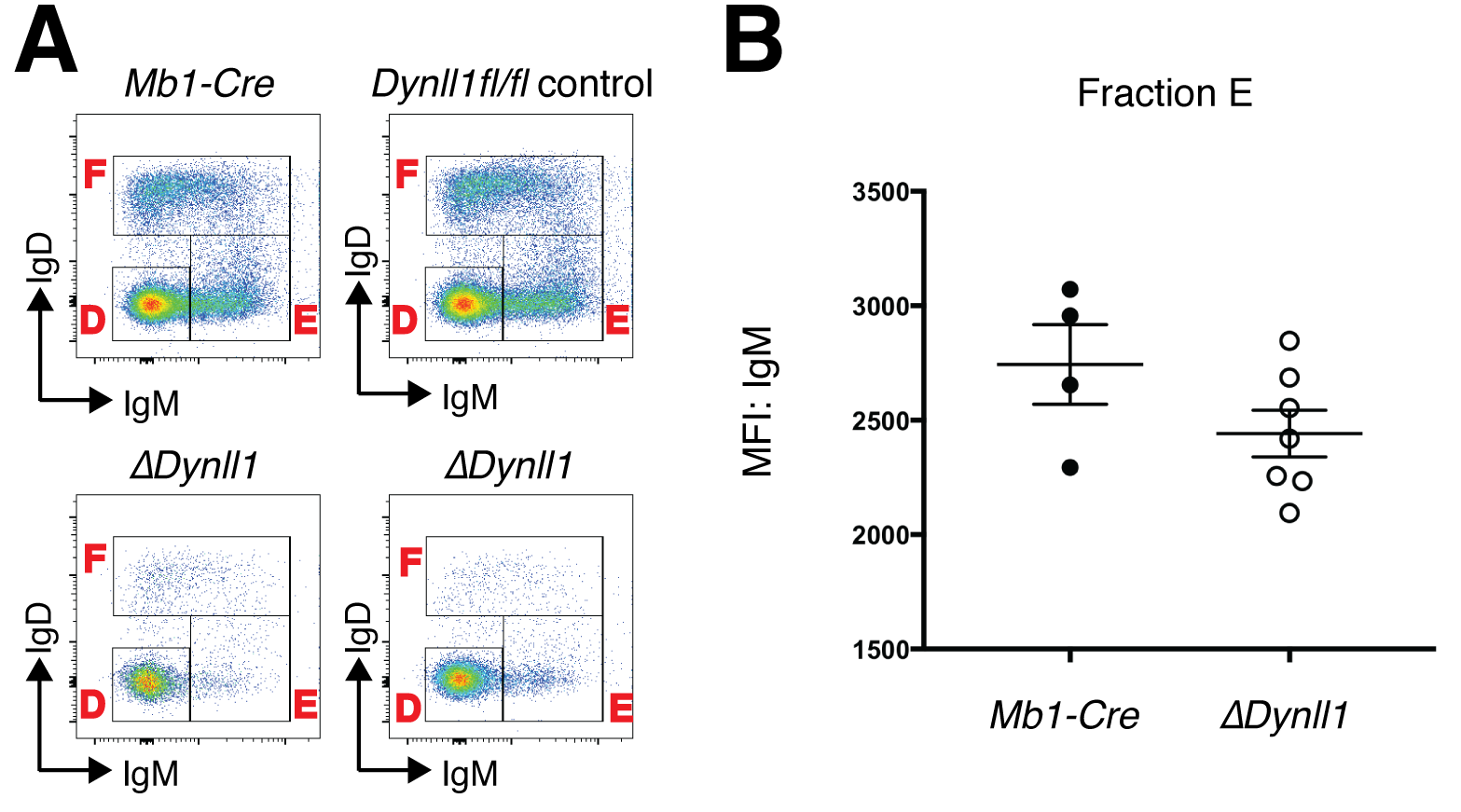

Supplement: S2 Fig — (A) FACS plots of B220+CD43- bone marrow cells from one Mb1-Cre control mouse, one Dynll1fl/fl control mouse and two Dynll1-deleted mice with fractions D (small pre-B), E (immature) and F (recirclating) highlighted. (B) Mean fluorescence intensity (M.F.I.) of the IgM signal in fraction E cells (mean ± S.E.). (TIF) [file pgen.1007010.s002.tif]

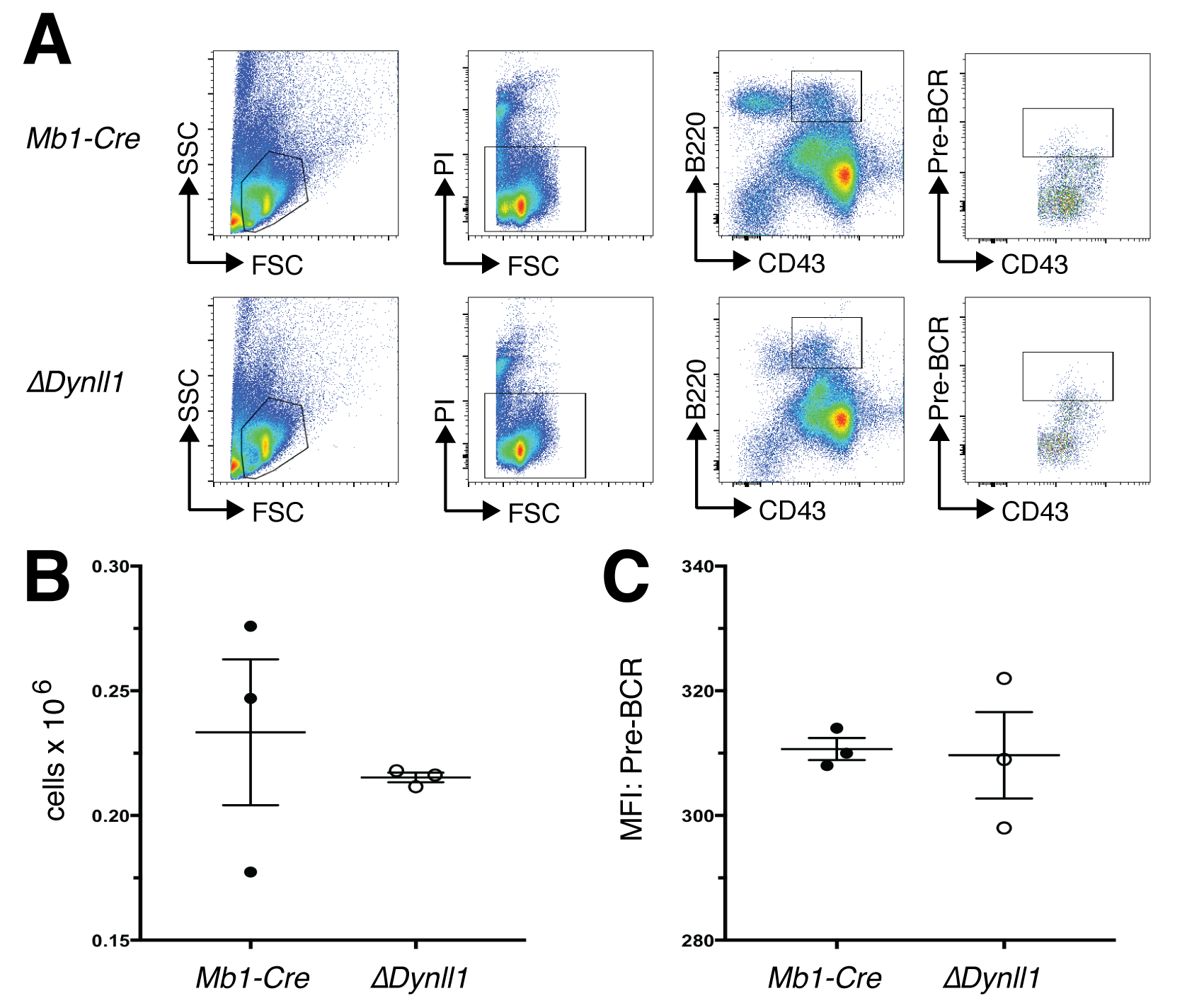

Supplement: S3 Fig — (A, B) Representative FACS plots and gating strategies to determine pre-BCR (SL156) expression on bone marrow pre-B lymphocytes (mean ± S.E.) in 8-to-10-week old mice. (C) Mean fluorescence intensity (M.F.I.) of the pre-BCR (SL156) signal on CD43+B220+ bone marrow lymphocytes (mean ± S.E.). (TIF) [file pgen.1007010.s003.tif]

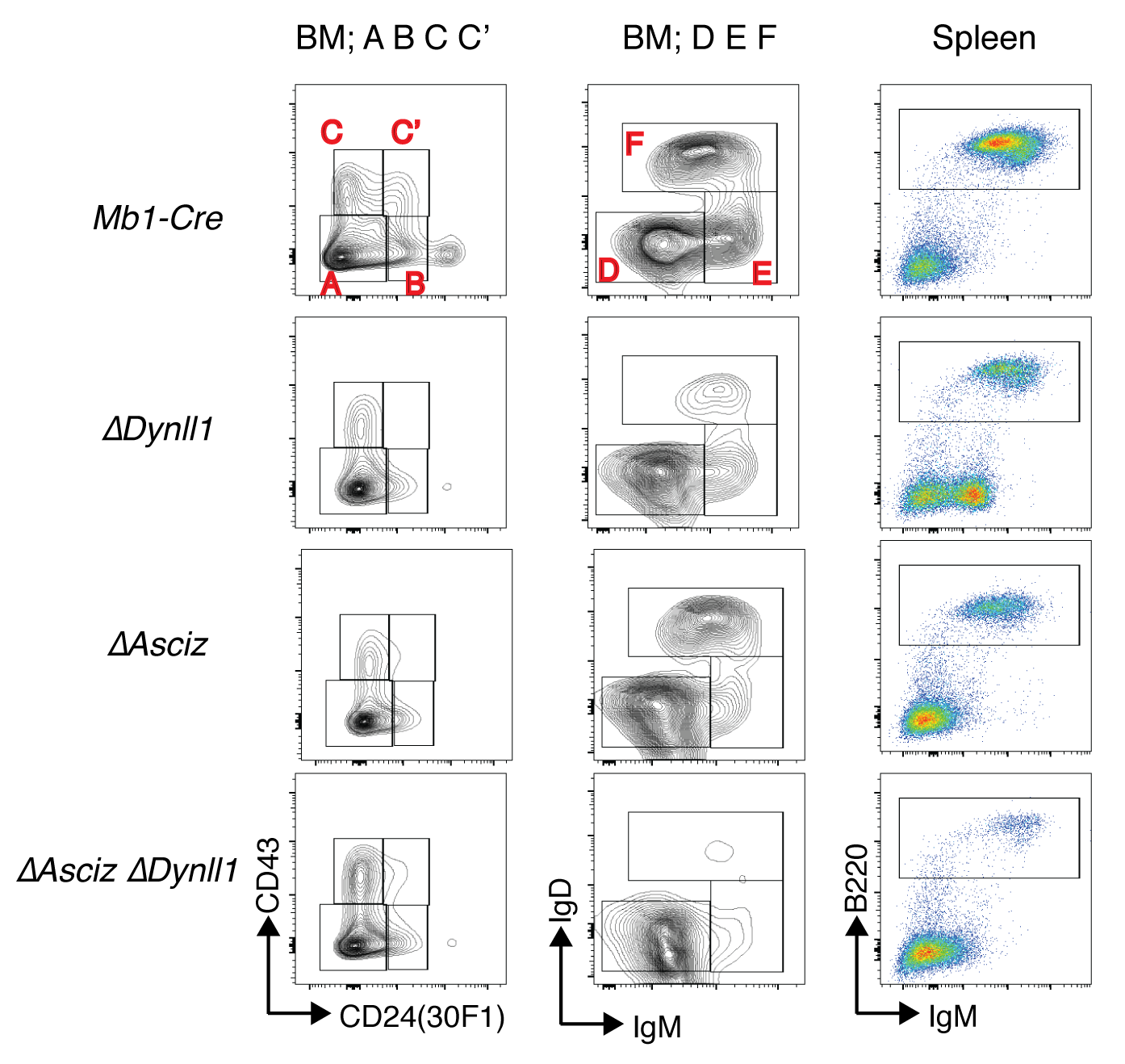

Supplement: S4 Fig — Representative FACS plots indicate gating strategies to determine total and mature splenic B cell numbers. (TIF) [file pgen.1007010.s004.tif]

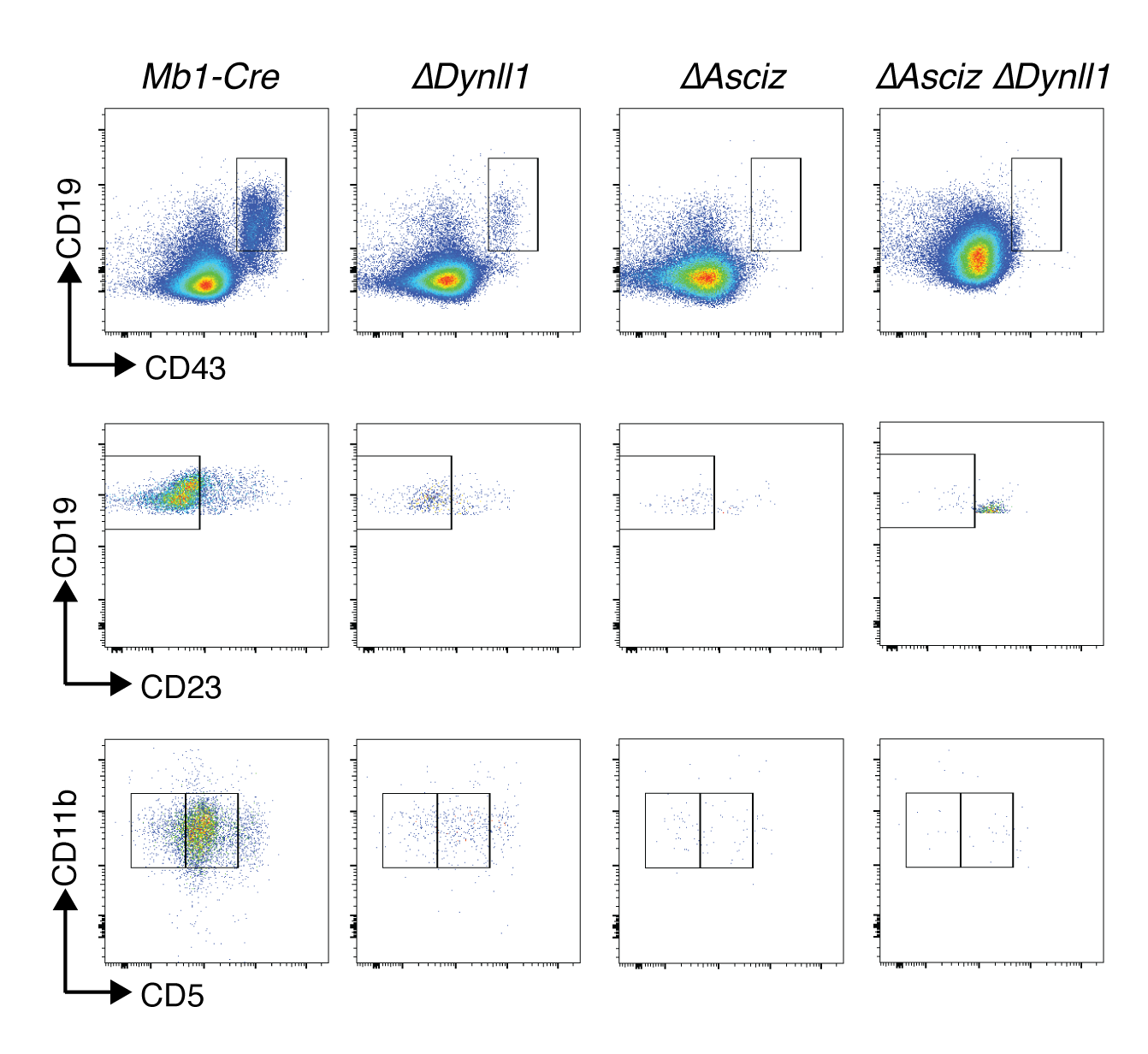

Supplement: S5 Fig — Representative FACS plots used for the quantification of B-1a cell numbers in the peritoneal cavity of 8-week old mice in Fig 7. (TIF) [file pgen.1007010.s005.tif]

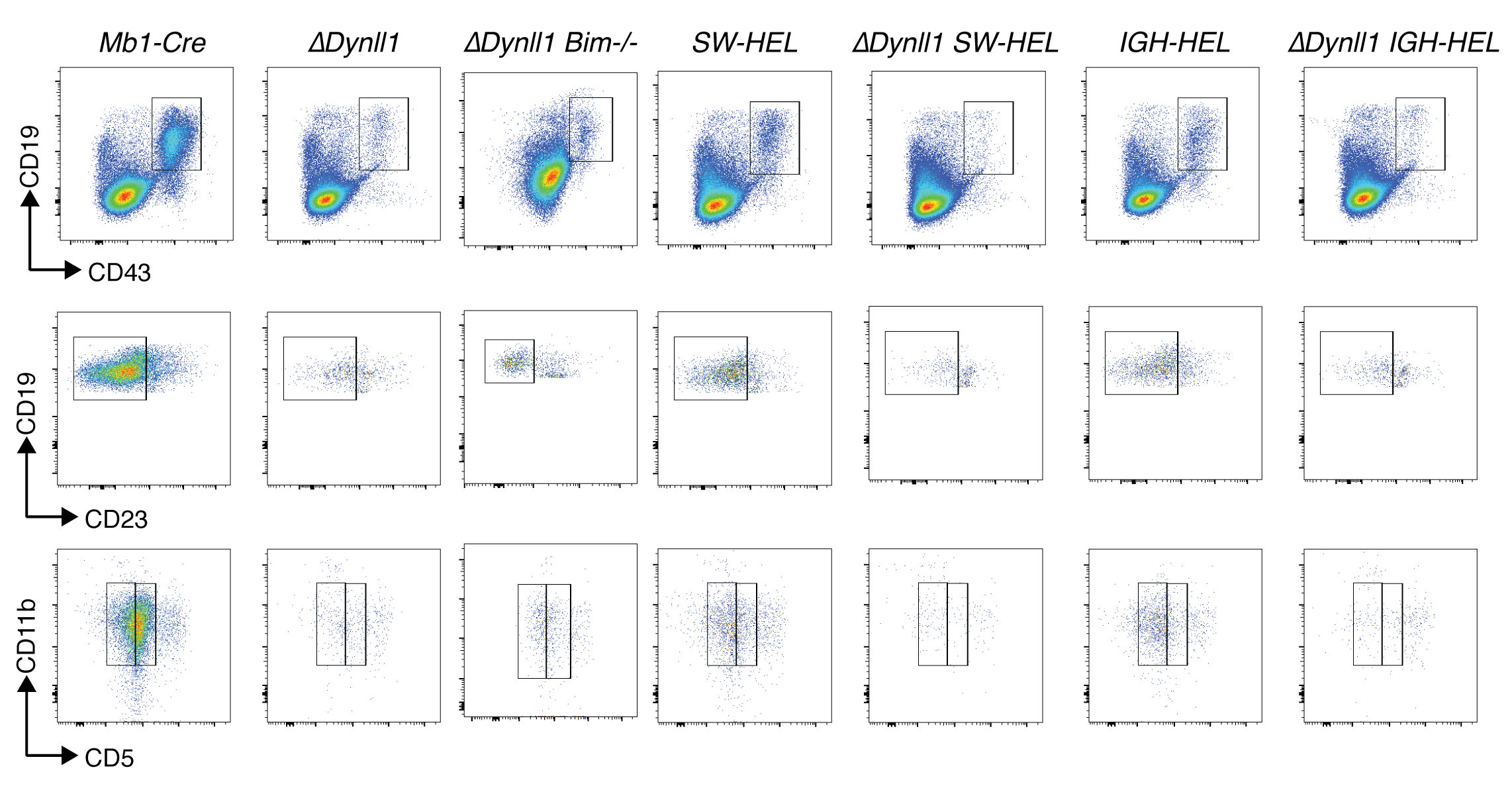

Supplement: S6 Fig — Representative FACS plots of peritoneal cavity cells of 8-week old mice related to the quantitative analyses in Fig 8. (TIF) [file pgen.1007010.s006.tif]

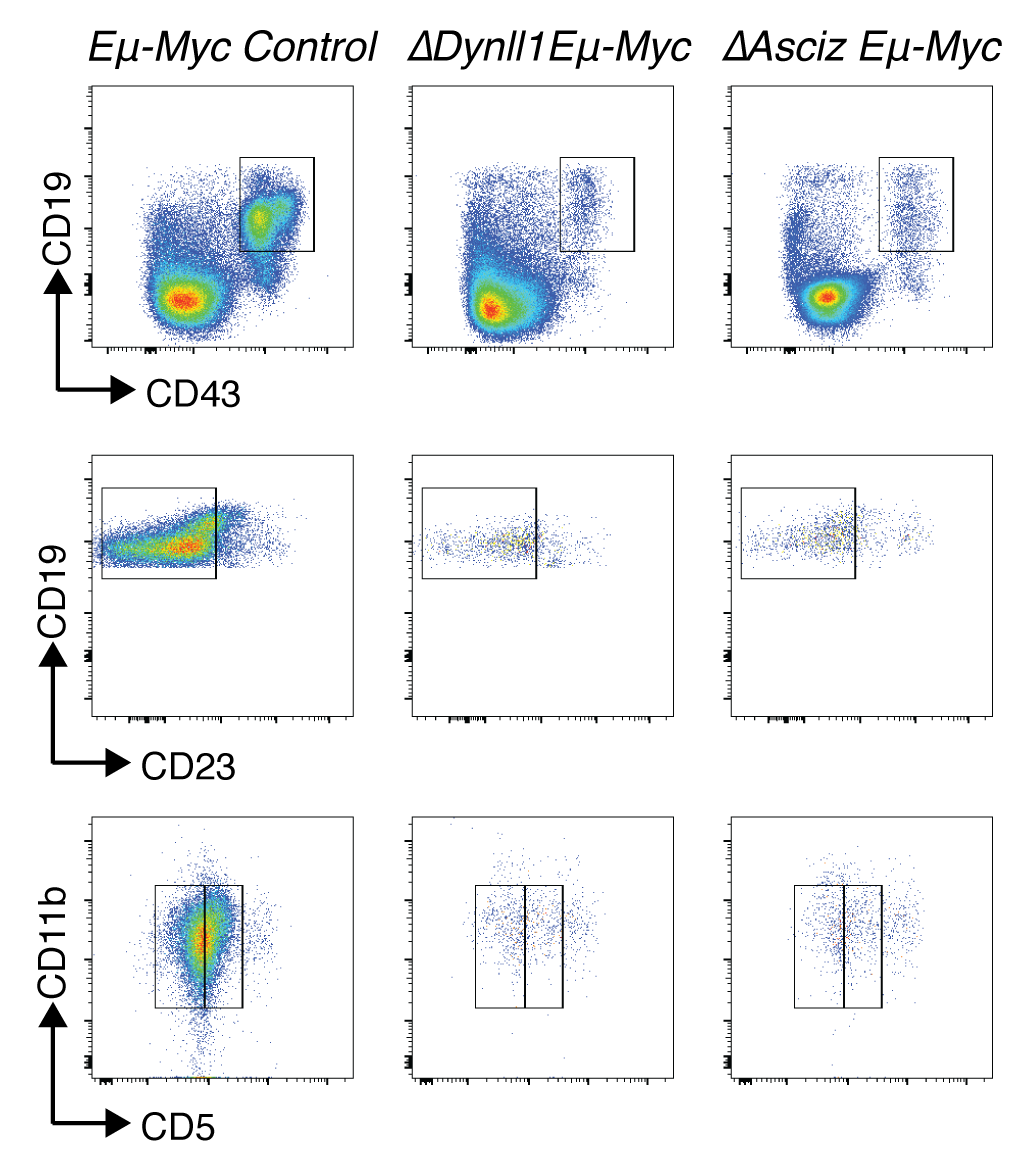

Supplement: S7 Fig — Representative FACS plots of peritoneal cavity cells of 8-week old mice, related to Fig 9A. (TIF) [file pgen.1007010.s007.tif]
